# Precision Oncology Insights into WNT Pathway Alterations in FOLFOX-Treated Early-Onset Colorectal Cancer in High-Risk Populations

**DOI:** 10.3390/cancers17172833

**Published:** 2025-08-29

**Authors:** Fernando C. Diaz, Brigette Waldrup, Francisco G. Carranza, Sophia Manjarrez, Enrique Velazquez-Villarreal

**Affiliations:** 1 Lineberger Comprehensive Cancer Center, University of North Carolina, Chapel Hill, NC 27514, USA; 2 Department of Integrative Translational Sciences, Beckman Research Institute, City of Hope, Duarte, CA 91010, USA; 3 City of Hope Comprehensive Cancer Center, Duarte, CA 91010, USA

**Keywords:** early-onset colorectal cancer, FOLFOX chemotherapy, genomics, ancestry, precision medicine, colorectal cancer, pharmacogenomics, cancer genetics

## Abstract

Background/Objectives: Early-onset colorectal cancer (EOCRC), defined as diagnosis before age 50, is rising rapidly and disproportionately affects Hispanic/Latino (H/L) populations. While FOLFOX is a standard first-line chemotherapy, its impact on tumor genomics in EOCRC remains poorly understood. Given the central role of WNT signaling in colorectal cancer (CRC), we aimed to characterize WNT pathway alterations in EOCRC across diverse populations and assess their associations with FOLFOX treatment and clinical outcomes. Methods: Somatic mutation data from 2515 CRC patients (266 H/L, 2249 Non-Hispanic White [NHW]) were analyzed. Patients were stratified by age (EOCRC vs. late-onset colorectal cancer), ancestry (H/L vs. NHW), and FOLFOX treatment status. Mutation frequencies in WNT pathway genes were compared, and Kaplan–Meier analysis evaluated overall survival. Results: WNT pathway alterations were pervasive across groups, with APC mutations dominating. Notably, non-canonical WNT mutations (e.g., CTNNB1, RNF43) were significantly less frequent in FOLFOX-treated H/L EOCRC patients compared to untreated individuals, suggesting potential chemotherapy-driven selection. In NHW patients, FOLFOX treatment was associated with reduced mutation frequencies across multiple WNT regulators, which correlated with improved overall survival. Conclusions: Our findings reveal that WNT pathway dysregulation in EOCRC is shaped by ancestry, treatment status, and age. FOLFOX appears to reduce specific WNT alterations in H/L patients and broader WNT disruptions in NHW patients, with survival benefits observed primarily in the latter. These results underscore ancestry-specific molecular responses to chemotherapy and the need for precision oncology strategies tailored to high-risk populations.

## Simple Summary

Colorectal cancer diagnosed before age 50, known as early-onset colorectal cancer, is rising quickly, especially in Hispanic and Latino individuals. A common chemotherapy called FOLFOX is often used to treat this cancer, but we know little about how it affects tumor biology in younger patients from different backgrounds. This study looked at changes in genes related to the WNT pathway, which plays a key role in colorectal cancer development. We found that certain gene changes were less common in patients who received FOLFOX, especially among Hispanic/Latino and Non-Hispanic White patients. These gene differences were also linked to differences in survival, particularly for Non-Hispanic White patients. Our findings suggest that chemotherapy may work differently depending on a patient’s background and highlight the importance of developing personalized treatment strategies for high-risk groups.

## 1. Introduction

Colorectal cancer (CRC) is the third most commonly diagnosed cancer in the United States and remains a leading cause of cancer-related deaths [1]. While overall incidence has declined in high-income nations due to improved screening [2], early-onset colorectal cancer (EOCRC), defined as diagnosis before age 50, has risen sharply [3,4,5]. Projections suggest EOCRC may become the leading cause of cancer-related death among individuals aged 20–49 by 2030 [4]. These increases affect all racial and ethnic groups but are especially pronounced in Hispanic/Latino (H/L), non-Hispanic Black, and American Indian/Alaska Native populations [6,7,8,9,10,11,12], underscoring both the rising burden and existing disparities in outcomes for high-risk groups.

Emerging evidence suggests EOCRC is biologically distinct from late-onset CRC (LOCRC), with differences in mutational landscapes and clinical behavior. Some reports describe elevated tumor mutation burden, microsatellite instability, or PD-L1 expression in EOCRC, though findings remain inconsistent [13,14,15]. Comparative genomic studies indicate alterations in multiple oncogenic pathways, including TP53, SMAD4, BRAF, NOTCH1, CTNNB1, APC, and KRAS [13,14,16], while epigenetic differences such as LINE-1 hypomethylation have also been proposed [17]. Collectively, these studies suggest EOCRC may harbor unique molecular features, though the clinical implications remain poorly defined, particularly in underserved populations where genomic data are limited.

Clinically, EOCRC is often diagnosed at advanced stages and associated with poorer survival [18,19]. For patients with metastatic, microsatellite stable (MSS), proficient mismatch repair (pMMR) CRC who lack targetable mutations, FOLFOX chemotherapy remains the standard of care [20,21]. However, recent reports suggest that younger patients may experience increased toxicity and reduced survival with FOLFOX compared to LOCRC patients [22]. These findings raise questions about whether specific genomic alterations influence treatment response in EOCRC.

Among the pathways implicated in CRC biology, WNT signaling plays a particularly central role. The pathway, commonly disrupted through mutations in APC, CTNNB1, and RNF43, regulates β-catenin activation and is altered in more than 80% of CRC tumors [23,24,25]. Intriguingly, although WNT pathway mutations appear less frequent in EOCRC, higher β-catenin activation has been observed [16,26]. The implications of this paradox, especially in the context of FOLFOX chemotherapy, remain unclear. Preclinical studies suggest WNT signaling may modulate responses to chemotherapy [27], but clinical evidence is lacking.

Given the rising burden of EOCRC in high-risk populations such as H/L individuals, and the limited understanding of how WNT alterations intersect with treatment outcomes, this study investigates the molecular landscape of WNT pathway dysregulation in MSS EOCRC treated with FOLFOX. By integrating genomic profiling with clinical outcome data, we aim to identify pathway-specific alterations that influence treatment response and highlight opportunities for precision oncology strategies tailored to underserved populations.

## 2. Materials and Methods

### 2.1. Clinical and Genomic Data

This study utilized clinical and genomic data from three publicly available CRC datasets curated through the cBioPortal for Cancer Genomics. To ensure the inclusion of detailed treatment information, datasets were selected based on the availability of chemotherapy and clinical annotation fields. The datasets included the following: Colorectal Adenocarcinoma (TCGA, PanCancer Atlas), MSK-CHORD and GENIE BPC CRC. Each dataset encompassed cases of colorectal, colon, and rectal adenocarcinomas. To maintain consistency, we restricted analysis to primary tumor samples and allowed only one tumor sample per patient.

Patients were identified as H/L based on annotations such as “Hispanic or Latino,” “Spanish, NOS”, “Hispanic, NOS”, or “Latino, NOS”. Individuals with Mexican or Spanish surnames were also included, following validated surname-based classification methods. The resulting cohort comprised 266 H/L patients (125 EOCRC and 141 LOCRC) and 2249 Non-Hispanic White (NHW) patients (677 EOCRC and 1572 LOCRC), all meeting identical inclusion criteria (Table 1 and Table 2). Age at diagnosis was extracted from clinical metadata. EOCRC was defined as diagnosis before age 50, while LOCRC was defined as diagnosis at age 50 or older.

To evaluate the role of treatment, cases were classified as “FOLFOX-treated” if they received a combination of leucovorin, fluorouracil (5-FU), and oxaliplatin administered within overlapping treatment timelines. Treatment data were manually curated from clinical drug administration tables provided in each dataset. Patients were further stratified by treatment status (FOLFOX-treated vs. non-treated), ethnicity (H/L vs. NHW), age group (EOCRC vs. LOCRC), and presence or absence of WNT pathway alterations.

The WNT pathway gene set analyzed in this study was curated from established CRC and WNT signaling literature [6,7,23,24,25,26,27]. Genes were included based on their well-characterized functional roles in canonical and non-canonical WNT signaling and their documented relevance to colorectal tumorigenesis. Specifically, this list encompasses key negative regulators of β-catenin stability (e.g., APC, AXIN1, AXIN2, AMER1, GSK3B), central effectors of pathway activation (e.g., CTNNB1, TCF7L2), and modulators of receptor activity (e.g., RNF43). Together, these genes represent the core molecular components most frequently disrupted in CRC and implicated in tumor initiation, progression, and therapeutic resistance. The complete list of WNT pathway genes analyzed is provided in Table S1.

Molecular alterations in the WNT pathway were defined using curated gene lists from the established CRC literature [6,7]. Genes with well-characterized roles in WNT signaling and colorectal tumorigenesis were included. Somatic alterations were extracted from cBioPortal and limited to nonsynonymous variants, including missense, nonsense, frameshift insertions or deletions, splice site mutations, and translation start site mutations. Pathway alteration burden was defined as the presence of at least one qualifying mutation in any WNT pathway member gene. Table 3 outlines the distribution of WNT pathway alterations by age, ethnicity, and treatment group. Table 3 further compares EOCRC cases between H/L and NHW patients stratified by treatment type, enabling an in-depth comparative analysis.

### 2.2. Statistical Analysis

To assess differences in mutation prevalence across demographic and treatment groups, chi-square (χ^2^) tests were conducted to evaluate associations between categorical variables, including age group, ethnicity, FOLFOX treatment status, and WNT pathway alteration status. When expected cell counts were <5, Fisher’s exact tests were employed to ensure robust statistical inference. Mutation rates for individual WNT pathway genes were calculated for each subgroup, and gene-by-gene comparisons were performed to identify significant differences in mutation frequency.

Tumor samples were also categorized by anatomical site (colon vs. rectal adenocarcinoma) to explore potential interactions between tumor location, WNT pathway status, and treatment response. Supplementary Tables S1 and S2 provide detailed gene-level mutation frequencies by age, ethnicity, and treatment type, supporting interpretation of molecular trends within and across subgroups.

Kaplan–Meier survival analyses were performed to determine the impact of WNT pathway alterations on overall survival in EOCRC and LOCRC patients, stratified by treatment and ethnicity. Survival curves were generated to visualize differences in survival probability over time between groups with and without WNT alterations. The log-rank test was used to assess statistical significance between survival distributions. Median survival times and 95% confidence intervals (CIs) were calculated to ensure accurate estimates. This multi-layered analytical framework provides a comprehensive evaluation of WNT pathway dysregulation in CRC, particularly in the context of FOLFOX treatment and underrepresented populations.

### 2.3. Study Design Overview

In summary, this study was designed to integrate clinical and genomic data from three large, publicly available CRC cohorts to evaluate the role of WNT pathway alterations in EOCRC across diverse populations. After curating treatment and demographic data, patients were stratified by age (EOCRC vs. LOCRC), ancestry (H/L vs. NHW), and treatment status (FOLFOX-treated vs. untreated). Mutation frequencies in WNT pathway genes were systematically compared across groups using chi-square or Fisher’s exact tests, and subgroup-specific patterns were identified. Finally, Kaplan–Meier survival analyses were performed to assess the prognostic significance of WNT pathway alterations in relation to treatment exposure. A schematic representation of the study workflow (Figure S1) outlines the overall design, from dataset selection to statistical and survival analyses, providing a visual roadmap for the investigation.

## 3. Results

### 3.1. Clinical and Demographic Characteristics of Hispanic/Latino and Non-Hispanic White CRC Cohorts

Using data from three cBioPortal projects with ethnicity and treatment annotations, we identified two study cohorts: 266 H/L patients and 2249 NHW patients with primary colorectal, colon, or rectal adenocarcinoma. Within the H/L cohort, 27.4% (n = 73) were EOCRC patients treated with FOLFOX, 34.2% (n = 91) were LOCRC patients treated with FOLFOX, 19.5% (n = 52) were EOCRC without FOLFOX treatment, and 18.8% (n = 50) were LOCRC without FOLFOX treatment. In the NHW cohort, a smaller proportion (16.7%, n = 375) included EOCRC patients treated with FOLFOX, while 40.9% (n = 919) were LOCRC treated with FOLFOX. The remainder included 13.4% (n = 302) EOCRC and 29.0% (n = 653) LOCRC patients not treated with FOLFOX.

The sex distribution was similar across groups, with males comprising 59.4% of the H/L cohort and 56.3% of the NHW cohort. Females accounted for 40.6% of H/L patients and 43.7% of NHW patients. All patients included in this analysis had primary tumors, with 100% of both H/L and NHW patients meeting this criterion.

Regarding cancer type, most patients were diagnosed with colon adenocarcinoma—61.7% in the H/L group and 59.0% in the NHW group. Rectal adenocarcinoma accounted for 24.1% of H/L cases and 28.7% of NHW cases, while colorectal adenocarcinoma (not otherwise specified) comprised 14.3% and 12.2%, respectively.

Stage at diagnosis varied between groups. Among H/L patients, 58.6% were diagnosed with stage 1–3 disease, while 40.6% were diagnosed at stage 4. A small proportion (0.8%) had missing or unreported stage data. In the NHW cohort, 55.0% of patients had stage 1–3 disease, 44.7% had stage 4, and only 0.4% lacked stage information.

Ethnicity sub-classification within the H/L cohort showed that the majority (86.5%) was labeled as Spanish NOS, Hispanic NOS, or Latino NOS. An additional 11.3% were identified as Mexican (including Chicano), and 2.2% were classified as Other Spanish/Hispanic. In contrast, all 2249 NHW patients (100%) were confirmed as non-Hispanic White, ensuring clear separation between cohorts for comparative genomic and treatment analyses.

These clinical and demographic characteristics highlight distinct patterns of early-onset disease, treatment exposure, and diagnostic stage between H/L and NHW patients. The higher proportion of EOCRC among H/L patients, along with differences in cancer type and staging, underscores the need for stratified molecular analyses to guide precision oncology strategies in underserved populations.

### 3.2. Comparative Genomic Analysis by Age and Ancestry

A comparative analysis of clinical and genomic characteristics revealed key differences in EOCRC versus LOCRC among H/L patients (Table 2A). EOCRC H/L patients had a significantly younger median age at diagnosis (42 years; IQR: 37–46) compared to LOCRC H/L patients (66 years; IQR: 58–73, *p* < 0.001). The median mutation burden was slightly lower in EOCRC (7 mutations) than in LOCRC (8 mutations), though not statistically significant (*p* = 0.21). Notably, CTNNB1 mutations were significantly more frequent in EOCRC H/L patients (9.6%) than in LOCRC H/L patients (2.1%, *p* < 0.05). APC mutations were more common in LOCRC (73.8%) than in EOCRC (62.4%), but this difference did not reach significance (*p* = 0.06). RNF43 mutations trended higher in EOCRC (12.0%) compared to LOCRC (8.5%), though the difference was not statistically significant (*p* = 0.33).

Among NHW patients (Table 2B), similar age-related patterns emerged. The median age at diagnosis for EOCRC was 42 years (IQR: 37–46), compared to 67 years (IQR: 59–75) for LOCRC (*p* < 0.001). Mutation burden was again slightly lower in EOCRC (median = 7) versus LOCRC (median = 8), without statistical significance (*p* = 0.18). APC mutations were significantly less frequent in EOCRC (67.1%) than in LOCRC (75.9%, *p* < 0.01). Conversely, CTNNB1 mutations were more common in EOCRC (7.4%) than LOCRC (2.8%, *p* < 0.01). RNF43 mutations were modestly enriched in EOCRC (6.7%) compared to LOCRC (4.3%), though this difference was not statistically significant (*p* = 0.08).

When comparing EOCRC patients across ancestry groups (Table 2C), the median age at diagnosis was identical (42 years; IQR: 37–46, *p* = 0.89), and mutation burdens were comparable (median = 7 in both groups, *p* = 0.98). However, RNF43 mutations were significantly more frequent in EOCRC H/L patients than in NHW patients (12.3% vs. 6.7%, *p* < 0.05), suggesting a potential ancestry-specific enrichment. CTNNB1 mutations were also higher in H/L (9.6%) than NHW (7.4%) patients, though the difference was not significant (*p* = 0.43). APC mutations were slightly less prevalent in EOCRC H/L patients (62.4%) compared to NHW patients (67.1%, *p* = 0.33).

Comparative genomic analyses revealed consistent age-related differences in CRC, with EOCRC patients diagnosed at a younger age and showing distinct WNT pathway patterns. CTNNB1 mutations were enriched in EOCRC across both H/L and NHW groups, while APC mutations were more frequent in LOCRC. Notably, RNF43 mutations were significantly higher in EOCRC H/L patients compared to their NHW counterparts, suggesting a potential ancestry-specific enrichment.

### 3.3. Prevalence of WNT Pathway Alterations by Age, Ancestry, and FOLFOX Treatment Status

An integrated analysis of WNT pathway alterations revealed a consistently high prevalence across all subgroups of CRC patients, regardless of age of onset, ancestry, or FOLFOX treatment status.

In the H/L cohort (Table 3A), WNT pathway alterations were present in 86.3% of EOCRC patients treated with FOLFOX and in 94.2% of untreated EOCRC cases, though this difference was not statistically significant (*p* = 0.23). Among LOCRC patients, 83.5% of FOLFOX-treated and 84.0% of untreated individuals harbored WNT alterations (*p* = 1.00). The proportion of patients without WNT alterations was slightly higher in treated EOCRC (13.7%) compared to untreated (5.8%), and nearly identical in LOCRC (16.5% vs. 16.0%). These findings indicate that WNT pathway dysregulation is widespread in H/L CRC, with minimal variation by age or chemotherapy exposure.

Similarly, in the NHW cohort (Table 3B), WNT alterations were detected in 84.3% of FOLFOX-treated EOCRC patients and 89.1% of untreated cases (*p* = 0.0889). Among LOCRC patients, prevalence was nearly identical between treated (86.2%) and untreated (87.0%) groups (*p* = 0.7009). Across all NHW subgroups, WNT alterations remained consistently high, and the proportion of patients without such alterations ranged from 10.9% to 15.7%, suggesting limited impact of FOLFOX on overall WNT mutational burden.

When comparing ancestry groups directly within the EOCRC population (Table 3C), the frequency of WNT alterations remained comparable between H/L and NHW patients. Among those treated with FOLFOX, 86.3% of H/L and 84.3% of NHW EOCRC patients had WNT pathway alterations (*p* = 0.7922). In untreated patients, 94.2% of H/L and 89.1% of NHW patients harbored such alterations (*p* = 0.3269). The proportion of patients without WNT alterations was low and similar across both groups.

Likewise, in the LOCRC subgroup (Table 3D), no significant differences were observed between H/L and NHW patients. WNT alterations were found in 83.5% of FOLFOX-treated H/L patients and 86.2% of their NHW counterparts (*p* = 0.5897), while the untreated group showed 84.0% and 87.0% prevalence, respectively (*p* = 0.7013).

Together, these findings demonstrate that WNT pathway alterations are highly prevalent across EOCRC and LOCRC, independent of ancestry or chemotherapy exposure. While specific gene-level differences may exist, the overall burden of WNT dysregulation appears to be a common and persistent feature of CRC biology in both H/L and NHW.

### 3.4. Frequencies of Gene Alterations in the WNT Pathway

#### 3.4.1. Gene-Level WNT Pathway Alterations in EOCRC H/L Patients by FOLFOX Treatment

To assess treatment-associated genomic differences, WNT pathway mutation frequencies were evaluated in EOCRC patients of H/L ancestry, stratified by FOLFOX exposure (Table S1). Notably, mutations in CTNNB1 and RNF43 were significantly more frequent in untreated patients (17.3% and 19.2%, respectively) compared to FOLFOX-treated patients (5.5% for both; *p* = 0.0405 and *p* = 0.0216). These findings suggest a potential selective effect of chemotherapy on non-canonical WNT components. In contrast, APC mutations were consistently high in both treated (80.8%) and untreated (84.6%) patients (*p* = 0.756), confirming its central role. Other genes (AMER1, AXIN1, AXIN2, GSK3B, TCF7L2) showed low mutation frequencies with no significant differences by treatment.

#### 3.4.2. Gene-Level WNT Pathway Alterations in LOCRC H/L Patients by FOLFOX Treatment

Among LOCRC H/L patients (Table S2), no statistically significant differences in WNT gene alterations were observed between FOLFOX-treated and untreated groups. APC mutations were the most frequent in both cohorts (71.4% vs. 72.0%; *p* = 1.00). RNF43 and AMER1 mutations showed slightly higher frequencies in treated patients (12.1% and 11.0%) compared to untreated patients (10.0% and 4.0%), though not statistically significant. Overall, FOLFOX did not appear to meaningfully impact the WNT mutation landscape in LOCRC H/L patients.

#### 3.4.3. Comparison Between EOCRC and LOCRC H/L Patients Treated with FOLFOX

In comparing WNT gene alterations between EOCRC and LOCRC H/L patients who received FOLFOX (Table S3), no significant differences were found. APC mutations were again the most prevalent (80.8% in EOCRC vs. 71.4% in LOCRC; *p* = 0.2266). Although RNF43 and AMER1 mutations were more common in LOCRC, these differences were not statistically significant. The distribution of CTNNB1, AXIN2, TCF7L2, and GSK3B mutations was similar across both age groups.

#### 3.4.4. Comparison Between Untreated EOCRC and LOCRC H/L Patients

Among untreated H/L patients (Table S4), RNF43 mutations were significantly more frequent in EOCRC than in LOCRC (19.2% vs. 10.0%; *p* = 0.0066). CTNNB1 mutations were also higher in EOCRC (17.3% vs. 4.0%), approaching significance (*p* = 0.0519). These findings point to a potential enrichment of non-canonical WNT pathway activity in younger patients. APC mutations were common across both groups. Other genes did not show statistically significant age-related differences.

#### 3.4.5. Gene-Level WNT Alterations in EOCRC NHW Patients by FOLFOX Treatment

In EOCRC NHW patients (Table S5), no significant differences in mutation frequencies were found between FOLFOX-treated and untreated individuals. APC mutations were the most frequent (77.6% vs. 81.1%; *p* = 0.304). CTNNB1, RNF43, and TCF7L2 mutations were observed at moderate levels, with no significant differences. Low-frequency mutations in AMER1, AXIN1, AXIN2, and GSK3B were consistent across treatment groups.

#### 3.4.6. Gene-Level WNT Alterations in LOCRC NHW Patients by FOLFOX Treatment

In LOCRC NHW patients (Table S6), FOLFOX treatment was associated with significantly lower mutation frequencies in several non-canonical WNT genes. AXIN1 (2.0% vs. 4.6%; *p* = 0.0045), AXIN2 (3.5% vs. 9.8%; *p* = 4.44 × 10^−7^), RNF43 (6.5% vs. 14.7%; *p* = 1.48 × 10^−7^), and TCF7L2 (13.1% vs. 18.1%; *p* = 0.0078) mutations were significantly depleted in treated patients. APC mutation frequencies were high in both groups and did not differ significantly.

#### 3.4.7. Comparison Between EOCRC and LOCRC NHW Patients Treated with FOLFOX

In NHW patients treated with FOLFOX (Table S7), no significant differences in WNT gene alterations were observed by age. APC mutations were the most frequent in both EOCRC (77.6%) and LOCRC (76.2%). Other gene alterations, including AMER1, CTNNB1, RNF43, and TCF7L2, occurred at similar rates, indicating a stable WNT mutational profile across age groups in treated NHW patients.

#### 3.4.8. Comparison Between EOCRC and LOCRC NHW Patients Not Treated with FOLFOX

Among untreated NHW patients (Table S8), several statistically significant differences were identified. APC mutations were significantly more common in EOCRC (81.1%) than in LOCRC (73.2%; *p* = 0.0100). In contrast, AXIN2 (9.8% vs. 5.3%; *p* = 0.0271) and RNF43 (14.7% vs. 6.6%; *p* = 5.66 × 10^−4^) mutations were enriched in LOCRC. These findings suggest age-related shifts in canonical versus non-canonical WNT signaling.

#### 3.4.9. Comparison Between EOCRC H/L and NHW Patients Treated with FOLFOX

Table S9 compares gene-level WNT alterations between FOLFOX-treated EOCRC patients of H/L and NHW ancestry. No significant differences were detected across the eight genes analyzed. APC, CTNNB1, and RNF43 mutation frequencies were nearly identical, suggesting a shared WNT mutational landscape between ancestries under similar treatment conditions.

#### 3.4.10. Comparison Between Untreated EOCRC H/L and NHW Patients

In contrast, comparison of untreated EOCRC patients (Table S10) revealed ancestry-associated differences. CTNNB1 mutations were significantly more frequent in H/L (17.3%) than NHW (7.6%; *p* = 0.0467), as were RNF43 mutations (19.2% vs. 6.6%; *p* = 0.0060). These findings point to enrichment of non-canonical WNT activation in H/L patients and suggest potential ancestry-specific molecular drivers.

#### 3.4.11. Comparison Between LOCRC H/L and NHW Patients Treated with FOLFOX

Among FOLFOX-treated LOCRC patients (Table S11), WNT mutation frequencies were generally similar between H/L and NHW groups. APC was the most frequently mutated gene in both (71.4% H/L vs. 76.2% NHW). While RNF43 mutations were more common in H/L patients (12.1% vs. 6.5%), this difference was not statistically significant (*p* = 0.0778).

#### 3.4.12. Comparison Between LOCRC H/L and NHW Patients Not Treated with FOLFOX

In untreated LOCRC patients (Table S12), no statistically significant ancestry-related differences were observed. However, AXIN2 mutations were more frequent in NHW patients (9.8%) compared to H/L (2.0%; *p* = 0.0748), suggesting possible ancestry-associated variation in non-canonical WNT signaling that warrants further study.

Gene-level analyses revealed that APC mutations were consistently the most frequent across all subgroups, confirming its central role in CRC. In EOCRC H/L patients, CTNNB1 and RNF43 mutations were significantly more common in untreated cases, suggesting potential chemotherapy-driven selection against these alterations. In NHW LOCRC patients, FOLFOX treatment was associated with significant reductions in several non-canonical WNT regulators (AXIN1, AXIN2, RNF43, TCF7L2). Comparisons by age and ancestry highlighted enrichment of non-canonical WNT alterations in untreated EOCRC and in H/L patients, pointing to potential ancestry- and age-specific drivers of pathway dysregulation.

### 3.5. Mutational Landscape

#### 3.5.1. Mutational Landscape of WNT Pathway in EOCRC H/L Patients

**Figure 1:**
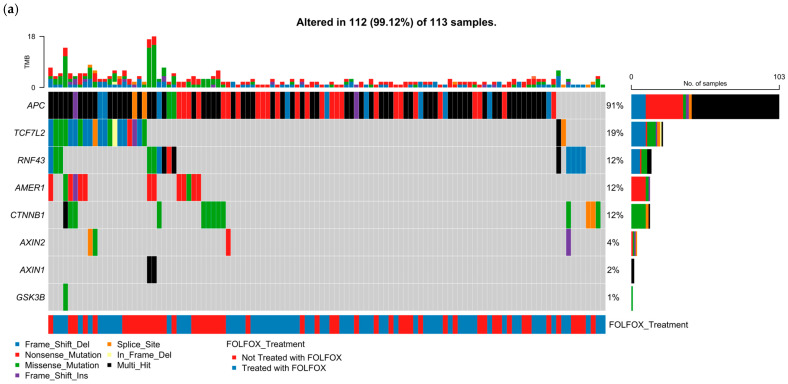

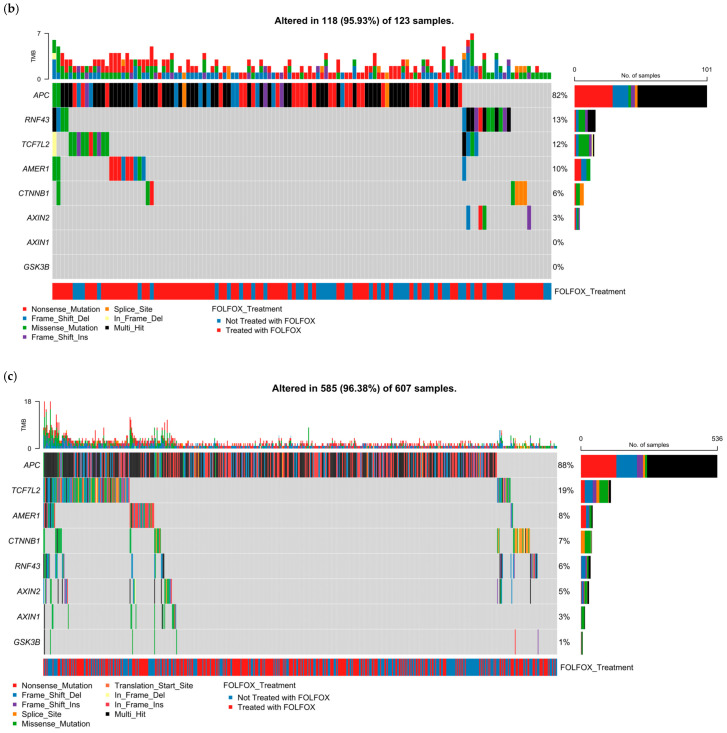

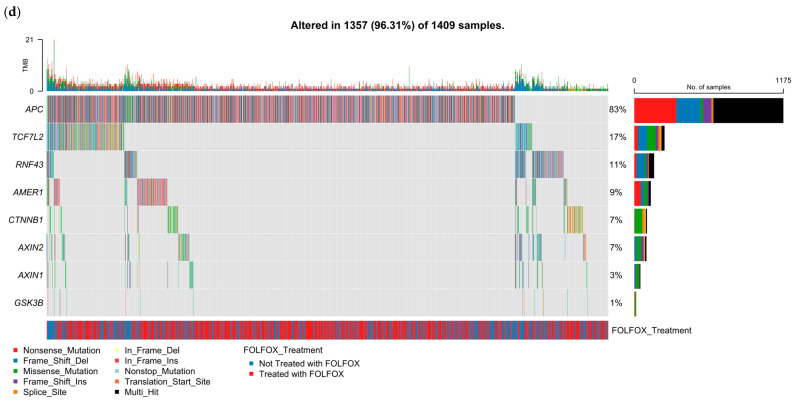
Somatic mutation landscape of WNT pathway genes in colorectal cancer (CRC) stratified by age and ancestry. Oncoplots displaying gene-level mutation profiles of WNT pathway components in colorectal cancer, stratified by age of onset (early vs. late) and ancestry (Hispanic/Latino vs. Non-Hispanic White). Panels show mutation types, tumor mutational burden (TMB), and FOLFOX treatment status across (**a**) 113 early-onset Hispanic/Latino (H/L) patients, (**b**) 123 late-onset H/L patients, (**c**) 607 early-onset Non-Hispanic White (NHW) patients, and (**d**) 1409 late-onset NHW patients. Across all subgroups, APC is the most frequently mutated gene, with truncating mutations predominating. Non-canonical WNT genes such as RNF43, TCF7L2, AMER1, and CTNNB1 exhibit additional recurrent alterations. The data highlight the pervasive disruption of WNT signaling in CRC and suggest age- and ancestry-associated variation in the somatic mutation landscape.

To characterize the mutational landscape of WNT signaling in EOCRC among H/L patients, we analyzed somatic alteration types and FOLFOX treatment status using an oncoplot representation (Figure 1a) alongside detailed mutation classifications (Table S13). Among the 113 EO H/L CRC samples evaluated, 112 (99.1%) harbored at least one mutation in a WNT pathway gene, highlighting the pervasive involvement of this pathway in tumorigenesis in this population.

The most frequently mutated gene was APC, altered in 91% of cases. APC mutations were predominantly truncating, including nonsense mutations (52.2%), frame shift deletions (26.1%), and multi-hit events, consistent with its established role as a gatekeeper of WNT signaling. RNF43 mutations were present in 12% of samples and were similarly enriched for truncating alterations, with frame shift deletions (53.3%) and nonsense mutations (20.0%) comprising the majority.

Additional commonly mutated genes included TCF7L2 (19%), AMER1 (12%), and CTNNB1 (12%). TCF7L2 exhibited a diverse spectrum of mutation types, including missense, splice site, and frame shift alterations. AMER1 was enriched for nonsense and missense mutations, while CTNNB1 displayed primarily missense and in-frame deletions. Less frequently altered WNT regulators included AXIN2 (4%), AXIN1 (2%), and GSK3B (1%), with a predominance of missense mutations in AXIN1 (85.7%) and AXIN2 (75.0%).

Tumor mutational burden (TMB), shown in the top bar plot of Figure 1a, was generally low across the cohort, though a subset of tumors exhibited elevated TMB, potentially suggestive of mismatch repair deficiency or hypermutator phenotypes. The bottom annotation illustrates FOLFOX treatment status, showing no clear segregation of mutation types between treated and untreated patients.

Overall, these findings underscore a high prevalence of WNT pathway alterations in EO H/L CRC, with a dominant pattern of truncating mutations in APC and diverse disruptions across multiple WNT pathway components, supporting their central role in early-onset tumorigenesis within this high-risk population.

#### 3.5.2. WNT Pathway Alterations in LOCRC H/L Patients

To assess the mutational landscape of WNT signaling in LOCRC among H/L patients, we analyzed mutation types and treatment status using an oncoplot framework (Figure 1b), complemented by detailed classification of variant types (Table S13). Among 123 LO H/L CRC samples, 118 (95.9%) harbored at least one somatic alteration in a WNT pathway gene, reinforcing the persistent involvement of this pathway in colorectal tumorigenesis across the age spectrum.

APC was again the most frequently mutated gene, altered in 82% of samples. The mutation spectrum was dominated by nonsense mutations (49.3%), frame shift deletions (30.4%), and multi-hit events, underscoring its central role as a truncating tumor suppressor. RNF43, mutated in 13% of cases, displayed a more heterogeneous profile, comprising frame shift deletions (43.8%), nonsense mutations (25.0%), and missense mutations (25.0%), indicating both loss-of-function and potentially modulatory effects.

Additional frequently mutated genes included TCF7L2 (12%) and AMER1 (10%). TCF7L2 alterations encompassed a diverse array of missense, splice site, and frame shift mutations, while AMER1 was enriched for nonsense and missense events. CTNNB1 mutations were observed in 6% of samples and were largely composed of missense and in-frame deletions, consistent with potential functional impacts on β-catenin regulation. AXIN2 was altered in 3% of cases, with a shift toward missense mutations (62.5%) but also a modest representation of truncating mutations. AXIN1 and GSK3B did not exhibit detectable mutations in this cohort.

The TMB, shown in the top bar plot of Figure 1b, was generally low across the cohort, although a subset of samples displayed elevated TMB, possibly reflecting mismatch repair deficiency or hypermutation. The distribution of FOLFOX treatment status (bottom annotation) revealed no distinct clustering or enrichment of mutation types between treated and untreated patients.

Collectively, these results demonstrate that the WNT signaling axis remains a critical driver of tumorigenesis in LO H/L CRC, with APC truncating mutations as the hallmark event, and additional contributions from diverse alterations in both canonical and non-canonical pathway components. The similarities in mutational architecture between EO and LO H/L CRC suggest a shared molecular vulnerability within this population.

#### 3.5.3. WNT Pathway Alterations in EOCRC NHW Patients

To delineate the mutational architecture of the WNT signaling pathway in EOCRC among NHW patients, we analyzed mutation types and FOLFOX treatment status using an oncoplot representation (Figure 1c), supported by mutation classification data (Table S13). Among 607 EO NHW CRC samples, 585 (96.4%) harbored at least one somatic alteration in a WNT pathway gene, confirming the centrality of WNT dysregulation in EOCRC tumorigenesis in this population.

APC mutations were the most frequent, found in 88% of cases, with a predominance of nonsense mutations (56.6%), frame shift deletions (28.3%), and multi-hit events, consistent with its role as a truncating tumor suppressor and negative regulator of WNT signaling. TCF7L2, altered in 19% of samples, showed a broad spectrum of mutation types, including missense, splice site, frame shift deletions, and in-frame insertions, suggesting diverse functional consequences.

RNF43 mutations were present in 6% of samples, largely consisting of frame shift deletions (56.3%) with additional nonsense and missense events. Mutations in AMER1 (8%) and CTNNB1 (7%) were also observed, with AMER1 showing a mixture of nonsense and missense alterations, while CTNNB1 exhibited missense mutations and in-frame deletions that may influence β-catenin activity. AXIN2 was mutated in 5% of cases, with a relatively even distribution of missense (30.0%), frame shift deletion (30.0%), and nonsense mutations (30.0%), indicating a broader mutation spectrum compared to H/L EOCRC cases. AXIN1 (3%) and GSK3B (1%) mutations were infrequent and predominantly missense or truncating in nature.

The TMB, displayed in the top panel of Figure 1c, was generally low across the cohort, although a few samples showed elevated TMB suggestive of hypermutator phenotypes. The FOLFOX treatment status, annotated at the bottom, showed a balanced distribution of treated and untreated cases across mutation types, with no evident treatment-associated clustering.

Together, these findings demonstrate a high prevalence of APC truncating mutations and widespread involvement of other WNT regulators in EO NHW CRC. The broader diversity of mutation types, particularly in genes such as AXIN2 and TCF7L2, suggests a more heterogeneous disruption of WNT signaling in this population, reinforcing the pathway’s central role in EOCRC tumorigenesis.

#### 3.5.4. WNT Pathway Alterations in LOCRC NHW Patients

To characterize WNT signaling disruptions in LOCRC among NHW patients, we analyzed 1409 tumor samples using oncoplot visualization (Figure 1d) and mutation classification data (Table S13). Of these, 1357 (96.3%) harbored at least one somatic alteration in a WNT pathway gene, reinforcing the pathway’s critical role in LOCRC tumorigenesis in this population.

APC emerged as the most frequently altered gene, mutated in 83% of samples. The majority of APC alterations were truncating in nature, including nonsense mutations (59.5%), frame shift deletions (25.7%), and multi-hit events, consistent with biallelic inactivation of this key tumor suppressor. This mutational pattern mirrored those seen in both younger NHW and H/L patients, underscoring APC’s conserved role across demographic groups.

Other WNT pathway components also demonstrated recurrent alterations. TCF7L2 (17%) displayed a diverse mutation spectrum, including missense, splice site, frame shift, and in-frame insertion/deletion mutations, suggesting various functional disruptions. RNF43 was mutated in 11% of samples, with frame shift deletions (61.5%) as the predominant event, alongside nonsense and missense mutations—indicative of its tumor suppressor role through truncating alterations. AMER1 mutations occurred in 9% of samples and were primarily nonsense and missense types. CTNNB1 and AXIN2 were each mutated in 7% of samples; CTNNB1 mutations were mostly missense and in-frame deletions, implicating potential dysregulation of β-catenin activity, while AXIN2 exhibited a broader range of mutation types, including missense, frame shift, and splice site variants. AXIN1 mutations (3%) and GSK3B mutations (1%) were less common, with changes predominantly classified as missense or frame shift events. As with earlier-onset cases, AXIN1 and AXIN2mutations were largely missense in nature (87.5% and 70.0%, respectively), indicating a consistent alteration pattern across both age and ethnicity.

TMB, illustrated in the top panel of Figure 1d, was generally low to moderate, although a subset of samples exhibited elevated TMB, potentially associated with microsatellite instability or hypermutation phenotypes. FOLFOX treatment status, annotated in the lower panel, showed a relatively even distribution of treated and untreated patients across all mutation types, with no distinct clustering by therapy.

The WNT pathway remains a major target of genetic disruption in LOCRC among NHW patients. APC truncating mutations predominate, accompanied by alterations in several additional WNT regulators. The diversity yet consistency of these mutation patterns across demographic groups underscore the robustness of WNT pathway dysregulation as a central driver of colorectal carcinogenesis and reinforce its importance as a potential therapeutic target.

### 3.6. Survival Analysis

We evaluated the association between WNT pathway alterations and overall survival across CRC subgroups defined by age, ancestry, and FOLFOX treatment status using Kaplan–Meier analysis.

**Figure 2:**
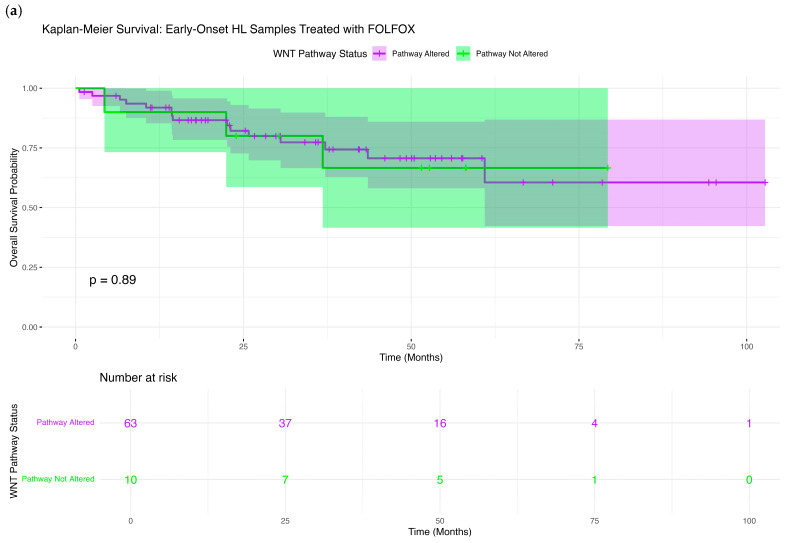

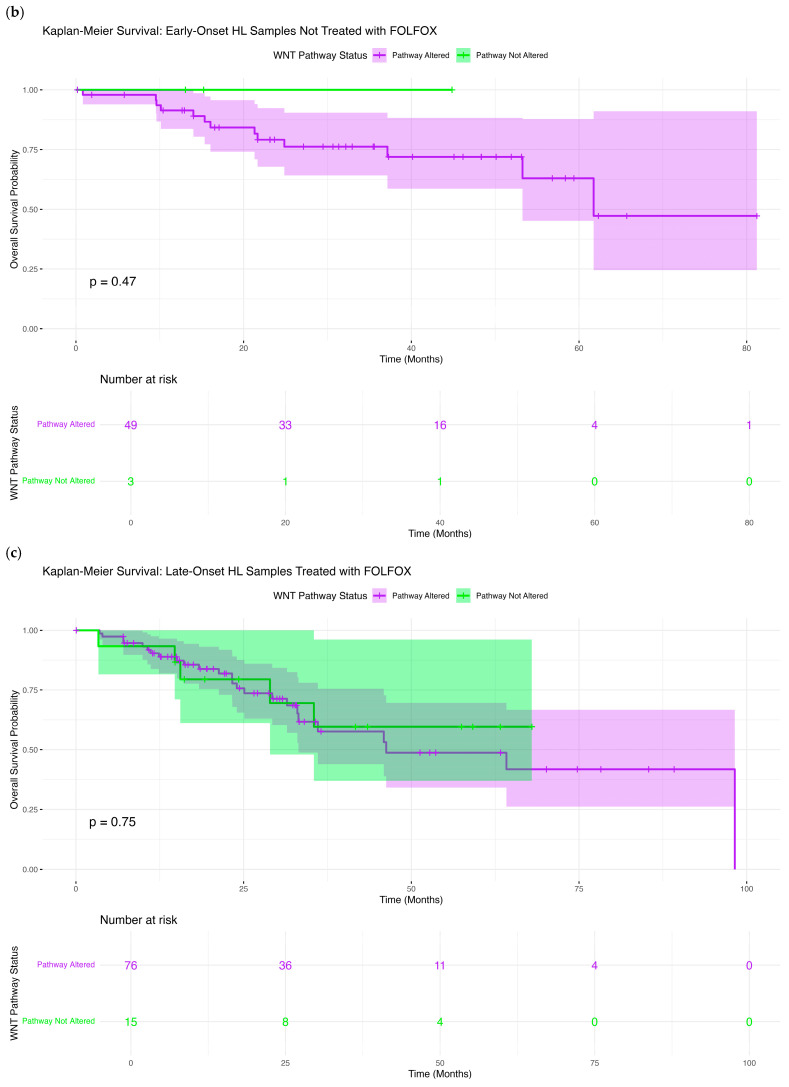

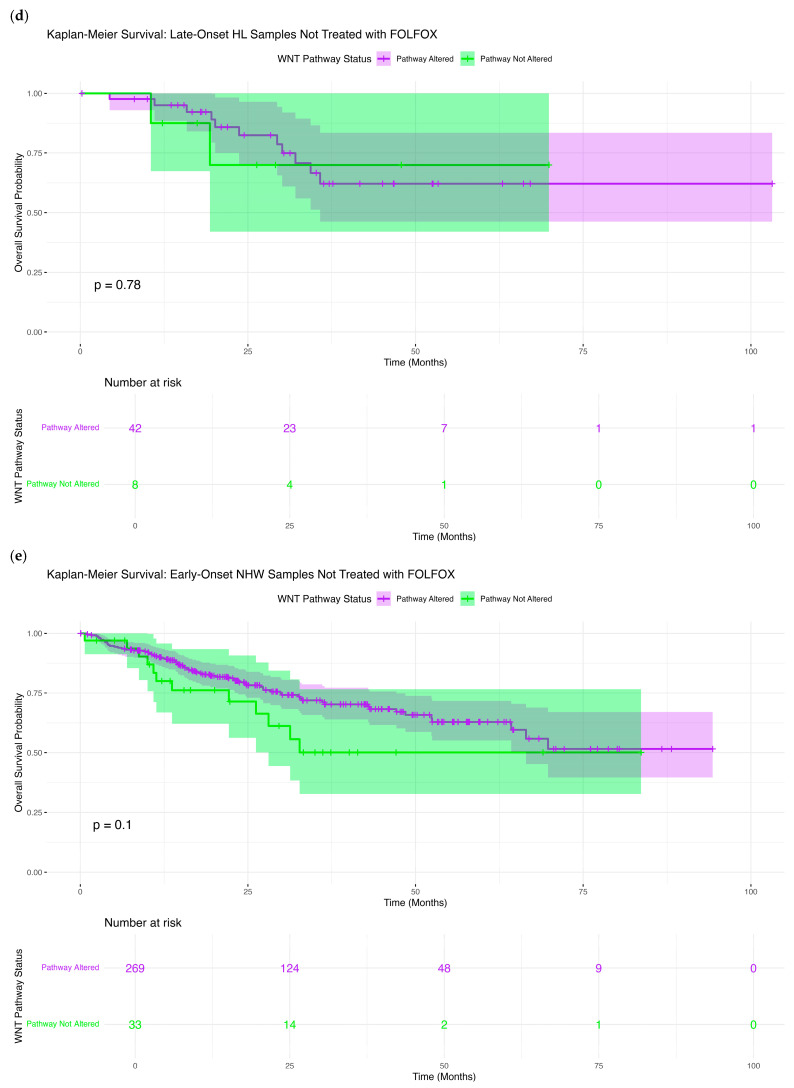

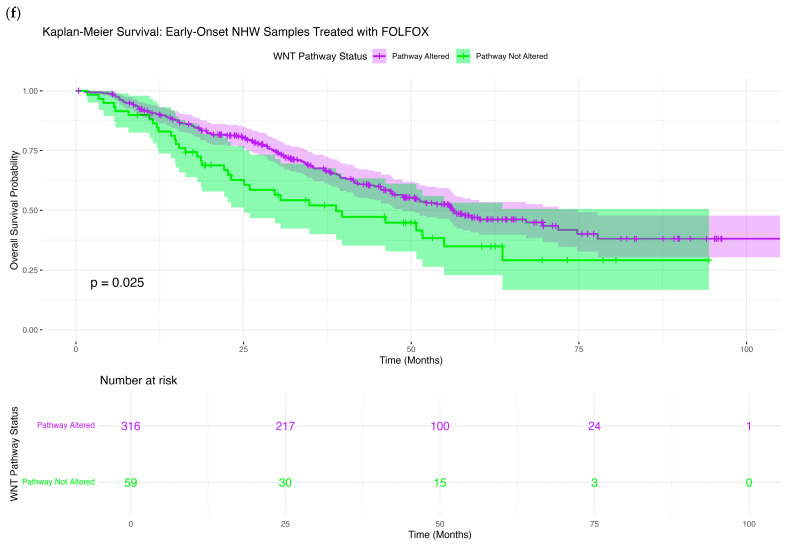
Comparative somatic mutation landscape of WNT pathway genes by age, ethnicity, and FOLFOX treatment status in colorectal cancer. Oncoplots illustrating mutation types and frequencies across WNT pathway genes in six colorectal cancer subgroups: (**a**) early-onset Hispanic/Latino (H/L) treated with FOLFOX, (**b**) early-onset H/L not treated with FOLFOX, (**c**) late-onset H/L treated with FOLFOX, (**d**) late-onset H/L not treated with FOLFOX, (**e**) early-onset Non-Hispanic White (NHW) treated with FOLFOX, (**f**) early-onset NHW not treated with FOLFOX.

Among H/L EOCRC patients treated with FOLFOX, no statistically significant difference in overall survival was observed between those with and without WNT pathway alterations (*p* = 0.89; Figure 2a). Survival trajectories were nearly identical across the follow-up period, with overlapping confidence intervals extending beyond 100 months. Although a minor dip in survival was noted for WNT-altered cases at intermediate time points, this difference diminished over time. Broad confidence intervals—particularly in the unaltered subgroup—reflect small sample sizes and contribute to uncertainty in the survival estimates.

Similarly, in H/L EOCRC patients not treated with FOLFOX, WNT alteration status did not significantly impact overall survival (*p* = 0.47; Figure 2b). Both groups showed comparable survival patterns, with a slight trend toward reduced survival in WNT-altered patients at later follow-up, though this was not statistically significant. Again, wide confidence intervals, especially in the unaltered group, limited interpretability.

For H/L LOCRC patients treated with FOLFOX, survival curves were largely overlapping regardless of WNT mutation status (*p* = 0.75; Figure 2c). While a modest drop in survival for WNT-altered patients was observed after 75 months, the difference was not statistically significant. Confidence intervals widened notably in later follow-up due to reduced numbers at risk.

In H/L LOCRC patients not treated with FOLFOX, no significant survival differences were detected between WNT-altered and unaltered groups (*p* = 0.78; Figure 2d). A brief early separation in curves favoring the unaltered group disappeared over time, with substantial widening of confidence intervals beyond 50 months, reflecting small sample sizes and increased uncertainty.

In contrast, among NHW EOCRC patients treated with FOLFOX, WNT pathway alterations were associated with significantly improved overall survival (*p* = 0.025; Figure 2e). Survival curves began diverging early, with WNT-altered patients showing higher survival probabilities that persisted throughout follow-up. Notably, by 50 months, the WNT-unmutated group experienced a sharper decline. Confidence intervals remained relatively narrow until late follow-up, where they widened due to fewer patients at risk.

For NHW EOCRC patients not treated with FOLFOX, no statistically significant survival difference was found (*p* = 0.1; Figure 2f). However, a trend toward poorer survival was observed in the WNT-unmutated group during the initial follow-up period. This early divergence diminished over time, and broad confidence intervals, particularly in the unaltered group, limited the strength of conclusions.

Among NHW LOCRC patients treated with FOLFOX, WNT pathway alterations were not significantly associated with overall survival (*p* = 0.42; Figure S2a). Although the WNT-unmutated group showed a slightly lower survival probability early on, curves converged later in follow-up. Confidence intervals widened notably beyond 50 months.

Finally, in NHW LOCRC patients not treated with FOLFOX, WNT pathway alterations were associated with significantly improved overall survival (*p* = 0.015; Figure S2b). WNT-altered patients maintained higher survival probabilities throughout the follow-up period, with early and persistent separation of survival curves. Confidence intervals remained relatively stable but widened at later time points due to declining numbers at risk.

## 4. Discussion

The rising incidence of EOCRC, particularly in high-risk populations such as H/L individuals in Southern California, demands a deeper understanding of the molecular drivers underlying disease onset and treatment response. This study represents one of the largest comparative analyses to date examining the genomic landscape of the WNT signaling pathway in microsatellite stable (MSS) EOCRC, with a specific focus on FOLFOX-treated patients across diverse racial/ethnic groups.

Our findings reveal consistently high frequencies of WNT pathway alterations in EOCRC across both H/L and NHW patients, underscoring the centrality of this pathway in early-onset tumorigenesis. APC mutations—primarily truncating—were the most prevalent across all subgroups, reaffirming their role as a foundational driver of WNT dysregulation. However, important differences emerged by age, treatment, and ancestry, suggesting that EOCRC is not a homogeneous disease and that WNT dysregulation may operate via distinct mechanisms in different patient populations.

Among H/L EOCRC patients, FOLFOX-treated tumors exhibited significantly lower frequencies of CTNNB1 and RNF43 mutations compared to untreated cases. This may reflect a selective pressure exerted by chemotherapy or potentially differential sensitivity of specific WNT alterations to FOLFOX-induced cytotoxicity. The reduced prevalence of these mutations following treatment warrants further investigation into whether CTNNB1 and RNF43 alterations confer treatment resistance or are preferentially eliminated by therapy. Conversely, untreated H/L EOCRC patients showed significantly higher frequencies of CTNNB1 and RNF43 mutations compared to their LOCRC counterparts, supporting the hypothesis that EOCRC in this population may be driven by non-canonical WNT activation.

In NHW patients, the mutation landscape also varied by age and treatment status. FOLFOX-treated LOCRC cases demonstrated significantly lower mutation rates in RNF43, AXIN1, AXIN2, and TCF7L2 compared to untreated counterparts, suggesting treatment-related selection or suppression of specific WNT-driven subclones. Interestingly, EOCRC NHW patients exhibited a distinct pattern with significantly lower APC and higher CTNNB1 mutation frequencies relative to LOCRC, further supporting the existence of age-associated divergence in WNT pathway disruption.

Despite the high prevalence of WNT alterations, survival analysis revealed that their prognostic implications were population-specific. In NHW patients, WNT pathway mutations were associated with improved survival in both FOLFOX-treated EOCRC and untreated LOCRC subgroups, suggesting potential value as predictive or prognostic biomarkers. In contrast, WNT alterations did not significantly affect survival outcomes in H/L patients, regardless of treatment or age group. These divergent findings may reflect underlying differences in tumor biology, treatment response, or unmeasured social determinants of health, and underscore the need for population-specific therapeutic strategies.

The diversity of mutation types—particularly in CTNNB1, RNF43, TCF7L2, and AMER1—further highlights the complexity of WNT signaling dysregulation. While APC truncations dominate, non-canonical regulators were variably altered across subgroups, pointing to alternate modes of pathway activation that may influence treatment sensitivity. The consistent prevalence of WNT alterations across treated and untreated patients suggests a foundational role in CRC pathogenesis, but the modulation of specific gene frequencies by FOLFOX raises the possibility of actionable gene–drug interactions.

In our stratified analysis of EOCRC patients of H/L ancestry, we found that CTNNB1 and RNF43 mutations were significantly enriched in untreated patients compared to those who received FOLFOX. This differential distribution raises the possibility that chemotherapy may selectively influence the prevalence of non-canonical WNT pathway alterations. One potential explanation is that tumors with CTNNB1 or RNF43 mutations may exhibit increased sensitivity to FOLFOX, resulting in a negative selection of these clones following treatment. Alternatively, such mutations may characterize a distinct biological subset of EOCRC with different therapeutic vulnerabilities. Importantly, APC mutations remained uniformly frequent in both treated and untreated patients, reaffirming its role as a core driver event in colorectal tumorigenesis. These observations highlight the potential interplay between chemotherapy and specific WNT pathway alterations and suggest that further mechanistic studies are warranted to explore whether CTNNB1 and RNF43 mutations could serve as biomarkers of response to FOLFOX in high-risk populations.

Several limitations should be considered when interpreting these results. First, the imbalance in sample sizes between H/L and NHW cohorts, as well as small subgroup numbers, reduces statistical power and may limit the generalizability of ancestry-specific findings. Second, reliance on multiple publicly available datasets introduces heterogeneity in sequencing platforms, clinical annotation, and treatment documentation, which could influence mutation frequency estimates and survival analyses. Third, missing clinical variables—such as comorbidities, detailed treatment regimens, and socioeconomic factors—restricts the ability to fully contextualize genomic findings within patient outcomes.

This study has important challenges. The retrospective design and reliance on publicly available datasets introduce potential biases in clinical annotations and treatment documentation. A marked imbalance in sample sizes between the H/L and NHW cohorts, along with small subgroup sizes, may reduce statistical power and affect the reliability of comparative and survival analyses, particularly in H/L patients. These findings should therefore be interpreted with caution and validated in larger, more balanced cohorts. Nonetheless, the use of well-annotated, multi-institutional datasets enabled robust subgroup analyses and highlighted critical differences that warrant confirmation in prospective studies.

Ancestry assignment in this study combined clinical annotations with validated surname-based classification methods to identify H/L patients. While surname algorithms have been widely used in population-based and genomic research to enhance representation of H/L populations, we acknowledge that this approach is not without limitations. Misclassification may occur in cases of intermarriage, adoption, or surnames shared across ethnic groups, potentially leading to either underestimation or overestimation of H/L cases. Nonetheless, prior studies have demonstrated reasonable accuracy and utility of surname-based methods, particularly when combined with clinical metadata, as was conducted here. Future studies that incorporate genomic ancestry markers alongside self-reported ethnicity and surnames will further improve the precision of population assignment in cancer disparities research.

While our study provides novel insights into WNT pathway alterations in EOCRC among H/L and NHW populations, it is important to acknowledge that the findings remain correlative in nature. Functional validation—such as assessing the impact of CTNNB1 or RNF43 mutations on FOLFOX sensitivity in preclinical models—was beyond the scope of this analysis but is necessary to determine whether these alterations directly mediate treatment response. The limited availability of datasets containing both genomic and detailed treatment information in underrepresented populations further constrained the scope of this study. Nonetheless, our analyses leverage one of the only comprehensive public resources that integrate clinical and molecular data in this context. Future efforts should focus on validating these results in prospective cohorts and experimental systems to better establish mechanistic links and guide the development of targeted precision oncology approaches.

Another important consideration is the need to validate these findings in broader and more diverse patient cohorts. While our study represents one of the largest available comparative analyses of WNT pathway alterations in EOCRC with treatment annotation, the relatively limited sample size—particularly among H/L patients—may restrict the generalizability of our results. Expanding analyses to larger, multi-ethnic populations and diverse clinical settings will be critical to determine whether the observed associations between WNT alterations and treatment response hold across different genetic backgrounds and tumor subtypes. In parallel, empirical testing of therapeutic strategies in preclinical models will be necessary to establish whether targeting components of the WNT pathway can overcome chemotherapy resistance. Such studies would provide an essential bridge from molecular insights to actionable interventions, ultimately informing precision oncology approaches tailored to high-risk populations.

Another important point is that the variability in dataset composition, clinical annotations, and sequencing methods introduces potential sources of heterogeneity that may influence outcomes. Our analyses therefore should be considered exploratory, providing hypotheses that require confirmation through standardized protocols and prospective studies. Future work incorporating harmonized clinical–genomic datasets, as well as functional validation in controlled experimental systems, will be essential to ensure reproducibility and strengthen the translational relevance of these findings. By acknowledging these limitations, we emphasize that while our study identifies important context-specific patterns in WNT pathway dysregulation, further validation is necessary to fully establish their reliability and clinical utility.

Although WNT pathway dysregulation is a well-established hallmark of CRC, our study advances the field by revealing how the mutational spectrum differs across demographic and treatment-defined subgroups. In particular, we demonstrate that while APC mutations remain the central driver across populations and treatment groups, alterations in non-canonical regulators such as CTNNB1 and RNF43 are significantly more frequent in untreated EOCRC patients of H/L ancestry. These findings suggest that chemotherapy may shape the prevalence of specific WNT pathway alterations and underscore the potential clinical relevance of considering ancestry and treatment history when interpreting genomic data. Thus, the take-home message of our work is that WNT pathway alterations are not only universal but also context-dependent, and this context—defined by patient ancestry, age, and treatment exposure—may hold important implications for tailoring precision oncology approaches in high-risk populations.

The emergence of next-generation artificial intelligence (AI) agents is poised to transform precision medicine by enabling real-time, integrative analysis of complex clinical and genomic data [28,29]. Platforms such as AI-HOPE (Artificial Intelligence for Health Outcomes and Precision Oncology) and AI-HOPE-PM (Artificial Intelligence agent for High-Optimization and Precision mEdicine in Population Metrics) represent a new paradigm in cancer research—facilitating hypothesis generation, pathway interrogation, and biomarker discovery through natural language-driven interfaces. Within this framework, specialized agents like AI-HOPE-WNT [30] have been developed to interrogate WNT signaling alterations by integrating multi-omics, treatment, and outcome data. Although AI tools were not applied in the present study, their future integration should be viewed as a complementary approach to the genomic analyses reported here. The identification of age-, ancestry-, and treatment-specific differences in WNT alterations underscores both the complexity of CRC biology and the challenges of interpreting heterogeneous, multi-institutional datasets—an area where AI-driven agents could provide significant value. By synthesizing diverse data sources and interrogating context-dependent patterns, such as the enrichment of CTNNB1 and RNF43 mutations in untreated EOCRC H/L patients, AI platforms offer the potential to generate hypotheses for further validation and extend the translational relevance of subgroup-specific findings. Situating AI-HOPE-WNT in this context highlights its role not as a separate focus but as a practical, forward-looking tool to accelerate the development of precision oncology strategies tailored to high-risk populations.

The observed differences in WNT alterations across age and ancestry groups may reflect underlying biological mechanisms that shape CRC pathogenesis. In younger patients, enrichment of CTNNB1 and RNF43 mutations suggests that non-canonical WNT activation may contribute more prominently to EOCRC, potentially driving more aggressive tumor biology and earlier onset of disease. By contrast, the predominance of APC truncations in LOCRC supports a canonical mode of pathway disruption that may develop over longer tumor latency periods. Ancestry-related variation, such as the higher frequency of RNF43 mutations in untreated H/L EOCRC patients, could be influenced by differences in genetic background, environmental exposures, or host–tumor interactions, including inflammation or microbiome composition, which have been shown to modulate WNT signaling. Chemotherapy may further shape these landscapes by selectively eliminating clones harboring mutations in non-canonical regulators, as seen in the reduced prevalence of CTNNB1 and RNF43 alterations among FOLFOX-treated patients. Together, these findings suggest that age- and ancestry-specific tumor biology, combined with treatment-related selective pressures, contribute to distinct patterns of WNT dysregulation in CRC.

## 5. Conclusions

In summary, this study demonstrates that WNT pathway alterations are highly prevalent in EOCRC across ancestries and treatment contexts, but their distribution and clinical significance vary by age, ethnicity, and FOLFOX exposure. The differential mutation profiles observed in H/L patients—particularly in CTNNB1 and RNF43—highlight the need for ancestry-informed molecular profiling in EOCRC. Moreover, the association between WNT mutations and improved survival in NHW patients points to their potential utility as predictive biomarkers. These findings support the integration of genomic, demographic, and treatment data to refine precision oncology strategies for EOCRC, especially in underserved and high-risk populations.

## Figures and Tables

**Table 1:** Clinical and demographic profiles of Hispanic/Latino (H/L) and Non-Hispanic White (NHW) colorectal cancer (CRC) patients in relation to age at onset, FOLFOX treatment, tumor characteristics, and ethnicity.

Clinical Feature	H/L Cohort *n* (%)	NHW Cohort *n* (%)
Age Onset & Treatment		
Early-Onset (<50) Treated with FOLFOX	73 (27.4%)	375 (16.7%)
Late-Onset (≥50) Treated with FOLFOX	91 (34.2%)	919 (40.9%)
Early-Onset (<50) Not Treated with FOLFOX	52 (19.5%)	302 (13.4%)
Late-Onset (≥50) Not Treated with FOLFOX	50 (18.8%)	653 (29.0%)
Cancer Type		
Colon Adenocarcinoma	164 (61.7%)	1328 (59.0%)
Rectal Adenocarcinoma	64 (24.1%)	646 (28.7%)
Colorectal Adenocarcinoma	38 (14.3%)	275 (12.2%)
Sex		
Male	158 (59.4%)	1267 (56.3%)
Female	108 (40.6%)	982 (43.7%)
Sample Type		
Primary Tumor	266 (100.0%)	2249 (100.0%)
Stage at Diagnosis		
Stage 1–3	156 (58.6%)	1236 (55.0%)
Stage 4	108 (40.6%)	1005 (44.7%)
NA	2 (0.8%)	8 (0.4%)
MSI Type		
Stable	200 (75.2%)	1940 (86.3%)
Instable	21 (7.9%)	209 (9.3%)
Indeterminate	10 (3.8%)	57 (2.5%)
NA	35 (13.2%)	43 (1.9%)
Ethnicity		
Spanish NOS; Hispanic NOS, Latino NOS	230 (86.5%)	0 (0.0%)
Mexican (includes Chicano)	30 (11.3%)	0 (0.0%)
Hispanic or Latino	2 (0.8%)	0 (0.0%)
Other Spanish/Hispanic	1 (0.4%)	0 (0.0%)
Spanish surname only	3 (1.1%)	0 (0.0%)
Non-Spanish; Non-Hispanic	0 (0.0%)	2249 (100.0%)

**Table 2:** Comparative clinical and genomic characteristics across early-onset and late-onset colorectal cancer (CRC) patient cohorts. This table summarizes clinical and molecular differences in WNT pathway alterations and mutation burden across key subgroups: (A) early-onset colorectal cancer (EOCRC) vs. late-onset colorectal cancer (LOCRC) among Hispanic/Latino (H/L) patients; (B) EOCRC vs. LOCRC among Non-Hispanic White (NHW) patients; and (C) EOCRC comparisons between H/L and NHW patient cohorts. Each comparison highlights age at diagnosis, total mutation count, and frequencies of select WNT pathway gene alterations, stratified by ethnicity and age group.

**(A)**						
**Clinical Feature**	**Early-Onset** **Hispanic/Latino** **Treated with FOLFOX** ***n* (%)**	**Early-Onset** **Hispanic/Latino** **Not Treated with FOLFOX** ***n* (%)**	***p*-Value**	**Late-Onset** **Hispanic/Latino** **Treated with FOLFOX** ***n* (%)**	**Late-Onset** **Hispanic/Latino** **Not Treated with FOLFOX** ***n* (%)**	***p*-Value**
Median Diagnosis Age (IQR)	42 (36–47)	40 (34–43)	0.05411	59 (54–66)	62 (56–70)	0.04865
Median Mutation Count	7 (5–8)	7 (5–20)	0.09735	8 (6–9) [NA = 1]	7 (5.25–9)	0.6507
Median TMB (IQR)	6.3 (4.5–7.8) [NA = 15]	5.5 (3.4–8.3) [NA = 2]	0.1719	6.1 (4.9–7.8) [NA = 10]	6.9 (5.6–9.0) [NA = 2]	0.04389
Median FGA	0.18 (0.03–0.27) [NA = 6]	0.19 (0.03–0.29)	0.7661	0.15 (0.06–0.25) [NA = 7]	0.21 (0.04–0.3) [NA = 2]	0.5464
RNF43 Mutation						
Present	4 (5.5%)	10 (19.2%)	0.02157	11 (12.1%)	5 (10.0%)	0.9232
Absent	69 (94.5%)	42 (80.8%)		80 (87.9%)	45 (90.0%)	
CTNNB1Mutation						
Present	4 (5.5%)	9 (17.3%)	0.04052	5 (5.5%)	2 (4.0%)	1
Absent	69 (94.5%)	43 (82.7%)		86 (94.5%)	48 (96.0%)	
**(B)**						
**Clinical Feature**	**Early-Onset NHW** **Treated with FOLFOX** ***n* (%)**	**Early-Onset NHW** **Not Treated with FOLFOX** ***n* (%)**	***p*-Value**	**Late-Onset NHW** **Treated with FOLFOX** ***n* (%)**	**Late-Onset NHW** **Not Treated with FOLFOX** ***n* (%)**	***p*-Value**
Median Diagnosis Age (IQR)	43 (37–48)	44 (38–47)	0.5646	63 (57–69)	66 (57–74)	4.146 × 10^−7^
Median Mutation Count	6 (5–8) [NA = 4]	7 (5–9) [NA = 2]	0.1258	7 (5–9) [NA = 10]	8 (6–12) [NA = 3]	1.22 × 10^−5^
Median TMB (IQR)	5.7 (4.1–6.9)	5.7 (4.1–7.8)	0.4214	6.1 (4.3–8.2)	6.6 (4.9–10.4)	0.0002854
Median FGA	0.14 (0.04–0.24) [NA = 4]	0.15 (0.04–0.23) [NA = 2]	0.5589	0.16 (0.06–0.28) [NA = 6]	0.15 (0.05–0.27) [NA = 5]	0.1929
RNF43 Mutation						
Present	18 (4.8%)	20 (6.6%)	0.3919	60 (6.5%)	96 (14.7%)	1.479 × 10^−7^
Absent	357 (95.2%)	282 (93.4%)		859 (93.5%)	557 (85.3%)	
TCF7L2 Mutation						
Present	58 (15.5%)	60 (19.9%)	0.162	120 (13.1%)	118 (18.1%)	7.79 × 10^−3^
Absent	317 (84.5%)	242 (80.1%)		799 (86.9%)	535 (81.9%)	
**(C)**						
**Clinical Feature**	**Early-Onset** **Hispanic/Latino** **Treated with FOLFOX** ***n* (%)**	**Early-Onset NHW** **Treated with FOLFOX** ***n* (%)**	***p*-Value**	**Early-Onset** **Hispanic/Latino** **Not Treated with FOLFOX** ***n* (%)**	**Early-Onset NHW** **Not Treated with FOLFOX** ***n* (%)**	***p*-Value**
Median Diagnosis Age (IQR)	42 (36–47)	43 (37–48)	0.08467	40 (34–43)	44 (38–47)	0.0006016
Median Mutation Count	7 (5–8)	6 (5–8) [NA = 4]	0.942	7 (5–20)	7 (5–9) [NA = 2]	0.2601
Median TMB (IQR)	6.3 (4.5–7.8) [NA = 15]	5.7 (4.1–6.9)	0.05806	5.5 (3.4–8.3) [NA = 2]	5.7 (4.1–7.8)	0.5732
Median FGA	0.18 (0.03–0.27) [NA = 6]	0.14 (0.04–0.24) [NA = 4]	0.5556	0.19 (0.03–0.29)	0.15 (0.04–0.23) [NA = 2]	0.3612
CTNNB1 Mutation						
Present	4 (5.5%)	20 (5.3%)	1	9 (17.3%)	23 (7.6%)	0.04666
Absent	69 (94.5%)	355 (94.7%)		43 (82.7%)	279 (92.4%)	
RNF43 Mutation						
Present	4 (5.5%)	18 (4.8%)	0.7687	10 (19.2%)	20 (6.6%)	0.006037
Absent	69 (94.5%)	357 (95.2%)		42 (80.8%)	282 (93.4%)	

**Table 3:** Frequency of WNT pathway alterations in Hispanic/Latino colorectal cancer (CRC) patients stratified by age of onset and FOLFOX treatment status. This table summarizes the mutation frequencies of key genes involved in the WNT signaling pathway among Hispanic/Latino (H/L) CRC patients, stratified by (A) early-onset (EOCRC) vs. late-onset (LOCRC) and FOLFOX treatment status within the H/L cohort, (B) FOLFOX-treated vs. untreated patients within EOCRC and LOCRC subgroups, (C) EOCRC H/L vs. Non-Hispanic White (NHW) patients by FOLFOX treatment status, and (D) LOCRC H/L vs. NHW patients by FOLFOX treatment status. Genes analyzed include APC, CTNNB1, RNF43, AXIN1, AXIN2, AMER1, and TCF7L2. Statistically significant differences (*p* < 0.05, Chi-square or Fisher’s exact test) are indicated with asterisks. This stratified analysis highlights potential interactions between age, ancestry, chemotherapy exposure, and WNT pathway dysregulation. WNT pathway alterations were highly prevalent across all subgroups, with >80% of CRC patients affected regardless of age, ancestry, or FOLFOX treatment status. While minor differences in prevalence were observed, particularly between treated and untreated EOCRC patients, no statistically significant variation emerged at the pathway level, underscoring WNT dysregulation as a persistent hallmark of CRC biology.

**(A)**						
**Pathway Alterations**	**Early-Onset** **Hispanic/Latino** **Treated with FOLFOX** ***n* (%)**	**Early-Onset** **Hispanic/Latino** **Not Treated with FOLFOX** ***n* (%)**	***p*-Value**	**Late-Onset** **Hispanic/Latino** **Treated with FOLFOX** ***n* (%)**	**Late-Onset Hispanic/Latino** **Not Treated with FOLFOX** ***n* (%)**	***p*-Value**
WNT Alterations Present	63 (86.3%)	49 (94.2%)	0.2348	76 (83.5%)	42 (84.0%)	1
WNT Alterations Absent	10 (13.7%)	3 (5.8%)		15 (16.5%)	8 (16.0%)	
**(B)**						
**Pathway Alterations**	**Early-Onset NHW** **Treated with FOLFOX** ***n* (%)**	**Early-Onset NHW** **Not Treated with FOLFOX** ***n* (%)**	***p*-Value**	**Late-Onset NHW** **Treated with FOLFOX** ***n* (%)**	**Late-Onset NHW** **Not Treated with FOLFOX** ***n* (%)**	***p*-Value**
WNT Alterations Present	316 (84.3%)	269 (89.1%)	0.0889	792 (86.2%)	568 (87.0%)	0.7009
WNT Alterations Absent	59 (15.7%)	33 (10.9%)		127 (13.8%)	85 (13.0%)	
**(C)**						
**Pathway Alterations**	**Early-Onset** **Hispanic/Latino** **Treated with FOLFOX** ***n* (%)**	**Early-Onset NHW** **Treated with FOLFOX** ***n* (%)**	***p*-Value**	**Early-Onset** **Hispanic/Latino** **Not Treated with FOLFOX** ***n* (%)**	**Early-Onset NHW** **Not Treated with FOLFOX** ***n* (%)**	***p*-Value**
WNT Alterations Present	63 (86.3%)	316 (84.3%)	0.7922	49 (94.2%)	269 (89.1%)	0.3269
WNT Alterations Absent	10 (13.7%)	59 (15.7%)		3 (5.8%)	33 (10.9%)	
**(D)**						
**Pathway Alterations**	**Late-Onset** **Hispanic/Latino** **Treated with FOLFOX** ***n* (%)**	**Late-Onset NHW** **Treated with FOLFOX** ***n* (%)**	***p*-Value**	**Late-Onset Hispanic/Latino** **Not Treated with FOLFOX** ***n* (%)**	**Late-Onset NHW** **Not Treated with FOLFOX** ***n* (%)**	***p*-Value**
WNT Alterations Present	76 (83.5%)	792 (86.2%)	0.5897	42 (84.0%)	568 (87.0%)	0.7013
WNT Alterations Absent	15 (16.5%)	127 (13.8%)		8 (16.0%)	85 (13.0%)	

## Data Availability

All data used in the present study are publicly available at https://www.cbioportal.org/ (accessed on 18 March 2025) and https://genie.cbioportal.org (accessed on 18 March 2025). Additional data can be provided upon reasonable request to the authors.

## Supplementary Materials

The following supporting information can be downloaded at: https://www.mdpi.com/article/10.3390/cancers17172833/s1, Figure S1: Study Workflow Overview; Figure S2: Comparative Somatic Mutation Landscape of WNT Pathway Genes by Age, Ethnicity, and FOLFOX Treatment Status in Colorectal Cancer; Table S1: Early-Onset Hispanic/Latino Patients: Treated with FOLFOX vs. Not Treated with FOLFOX; Table S2: Late-Onset Hispanic/Latino Patients: Treated with FOLFOX vs. Not Treated with FOLFOX; Table S3: Early-Onset vs. Late-Onset Hispanic/Latino Patients: Treated with FOLFOX; Table S4: Early-Onset vs. Late-Onset Hispanic/Latino Patients: Not Treated with FOLFOX; Table S5: Early-Onset Non-Hispanic White Patients: Treated with FOLFOX vs. Not Treated with FOLFOX; Table S6: Late-Onset Non-Hispanic White Patients: Treated with FOLFOX vs. Not Treated with FOLFOX; Table S7: Early-Onset vs. Late-Onset Non-Hispanic White Patients: Treated with FOLFOX; Table S8: Early-Onset vs. Late-Onset Non-Hispanic White Patients: Not Treated with FOLFOX; Table S9: Early-Onset Hispanic/Latino vs. Early-Onset Non-Hispanic White Patients: Treated with FOLFOX; Table S10: Early-Onset Hispanic/Latino vs. Early-Onset Non-Hispanic White Patients: Not Treated with FOLFOX; Table S11: Late-Onset Hispanic/Latino vs. Late-Onset Non-Hispanic White Patients: Treated with FOLFOX; Table S12: Late-Onset Hispanic/Latino vs. Late-Onset Non-Hispanic White Patients: Not Treated with FOLFOX; Table S13: Mutation Spectrum of WNT Pathway Genes by Ancestry, Age of Onset, and FOLFOX Treatment Status in Colorectal Cancer.
