# Supplementary material for: Precision Oncology Insights into WNT Pathway Alterations in FOLFOX-Treated Early-Onset Colorectal Cancer in High-Risk Populations
Source: Cancers (Basel). 2025 Aug 29;17(17):2833. doi: 10.3390/cancers17172833 (PMC12427346; doi:10.3390/cancers17172833)
Supplement: Supplementary file 1 [file cancers-17-02833-s001.zip › cancers-3826883-supplementary.pdf]

Precision Oncology Insights into WNT Pathway Alterations in FOLFOX-Treated Early-Onset Colorectal Cancer in High-Risk Populations

Fernando C. Diaz, M.D. [1], Brigette Waldrup, B.S. [2], Francisco G. Carranza, Ph.D. [2], Sophia Manjarrez, B.S. [2] and Enrique Velazquez-Villarreal, M.D., Ph.D., M.P.H., M.S. [2,3]*

*Correspondence: Correspondence: evelazquezvilla@coh.org

Supplementary Materials:

Table S1. – Early-Onset Hispanic/Latino Patients: Treated with FOLFOX vs. Not Treated with FOLFOX.

WNT Pathway			
**Gene**	**Early-Onset Hispanic/Latino Treated with FOLFOX n (%)**	**Early-Onset Hispanic/Latino Not Treated with FOLFOX n (%)**	**p-value**
**AMER1 Mutation**			
Present	5 (6.8%)	8 (15.4%)	0.2136
Absent	68 (93.2%)	44 (84.6%)	
**APC Mutation**			
Present	59 (80.8%)	44 (84.6%)	0.756
Absent	14 (19.2%)	8 (15.4%)	
**AXIN1 Mutation**			
Present	0 (0.0%)	2 (3.8%)	0.416
Absent	73 (100.0%)	50 (96.2%)	
**AXIN2 Mutation**			
Present	3 (4.1%)	1 (1.9%)	0.6404
Absent	70 (95.9%)	51 (98.1%)	
**CTNNB1 Mutation**			
Present	4 (5.5%)	9 (17.3%)	**0.04052**
Absent	69 (94.5%)	43 (82.7%)	
**GSK3B Mutation**			
Present	1 (1.4%)	0 (0.0%)	1
Absent	72 (98.6%)	52 (100.0%)	
**RNF43 Mutation**			
Present	4 (5.5%)	10 (19.2%)	**0.02157**
Absent	69 (94.5%)	42 (80.8%)	
**TCF7L2 Mutation**			
Present	11 (15.1%)	11 (21.2%)	0.5207
Absent	62 (84.9%)	41 (78.8%)	

Table S2 – Late-Onset Hispanic/Latino Patients: Treated with FOLFOX vs. Not Treated with FOLFOX

WNT Pathway			
**Gene**	**Late-Onset Hispanic/Latino Treated with FOLFOX n (%)**	**Late-Onset Hispanic/Latino Not Treated with FOLFOX n (%)**	**p-value**
**AMER1 Mutation**			
Present	10 (11.0%)	2 (4.0%)	0.2132
Absent	81 (89.0%)	48 (96.0%)	
**APC Mutation**			
Present	65 (71.4%)	36 (72.0%)	1
Absent	26 (28.6%)	14 (28.0%)	
**AXIN1 Mutation**			
Present	0 (0.0%)	0 (0.0%)	1
Absent	91 (100.0%)	50 (100.0%)	
**AXIN2 Mutation**			
Present	3 (3.3%)	1 (2.0%)	1
Absent	88 (96.7%)	49 (98.0%)	
**CTNNB1 Mutation**			
Present	5 (5.5%)	2 (4.0%)	1
Absent	86 (94.5%)	48 (96.0%)	
**GSK3B Mutation**			
Present	0 (0.0%)	0 (0.0%)	1
Absent	91 (100.0%)	50 (100.0%)	
**RNF43 Mutation**			
Present	11 (12.1%)	5 (10.0%)	0.9232
Absent	80 (87.9%)	45 (90.0%)	
**TCF7L2 Mutation**			
Present	9 (9.9%)	6 (12.0%)	0.9178
Absent	82 (90.1%)	44 (88.0%)	

Table S3 – Early-Onset vs. Late-Onset Hispanic/Latino Patients: Treated with FOLFOX

WNT Pathway			
**Gene**	**Early-Onset Hispanic/Latino Treated with FOLFOX n (%)**	**Late-Onset Hispanic/Latino Treated with FOLFOX n (%)**	**p-value**
**AMER1 Mutation**			
Present	5 (6.8%)	10 (11.0%)	0.5212
Absent	68 (93.2%)	81 (89.0%)	
**APC Mutation**			
Present	59 (80.8%)	65 (71.4%)	0.2266
Absent	14 (19.2%)	26 (28.6%)	
**AXIN1 Mutation**			
Present	0 (0.0%)	0 (0.0%)	1
Absent	73 (100.0%)	91 (100.0%)	
**AXIN2 Mutation**			
Present	3 (4.1%)	3 (3.3%)	1
Absent	70 (95.9%)	88 (96.7%)	
**CTNNB1 Mutation**			
Present	4 (5.5%)	5 (5.5%)	1
Absent	69 (94.5%)	86 (94.5%)	
**GSK3B Mutation**			
Present	1 (1.4%)	0 (0.0%)	0.4451
Absent	72 (98.6%)	91 (100.0%)	
**RNF43 Mutation**			
Present	4 (5.5%)	11 (12.1%)	0.1788
Absent	69 (94.5%)	80 (87.9%)	
**TCF7L2 Mutation**			
Present	11 (15.1%)	9 (9.9%)	0.443
Absent	62 (84.9%)	82 (90.1%)	

Table S4 – Early-Onset vs. Late-Onset Hispanic/Latino Patients: Not Treated with FOLFOX

WNT Pathway			
**Gene**	**Early-Onset Hispanic/Latino Not Treated with FOLFOX n (%)**	**Late-Onset Hispanic/Latino Not Treated with FOLFOX n (%)**	**p-value**
**AMER1 Mutation**			
Present	8 (15.4%)	2 (4.0%)	0.09268
Absent	44 (84.6%)	48 (96.0%)	
**APC Mutation**			
Present	44 (84.6%)	36 (72.0%)	0.1909
Absent	8 (15.4%)	14 (28.0%)	
**AXIN1 Mutation**			
Present	1 (1.9%)	0 (0.0%)	1
Absent	51 (98.1%)	50 (100.0%)	
**AXIN2 Mutation**			
Present	1 (1.9%)	1 (2.0%)	1
Absent	51 (98.1%)	49 (98.0%)	
**CTNNB1 Mutation**			
Present	9 (17.3%)	2 (4.0%)	**0.05193**
Absent	43 (82.7%)	48 (96.0%)	
**GSK3B Mutation**			
Present	0 (0.0%)	0 (0.0%)	1
Absent	52 (100.0%)	50 (100.0%)	
**RNF43 Mutation**			
Present	10 (19.2%)	5 (10.0%)	**0.006608**
Absent	42 (80.8%)	45 (90.0%)	
**TCF7L2 Mutation**			
Present	11 (21.2%)	6 (12.0%)	0.3299
Absent	41 (78.8%)	44 (88.0%)	

Table S5 – Early-Onset Non-Hispanic White Patients: Treated with FOLFOX vs. Not Treated with FOLFOX

WNT Pathway			
**Gene**	**Early-Onset NHW Treated with FOLFOX n (%)**	**Early-Onset NHW Not Treated with FOLFOX n (%)**	**p-value**
**AMER1 Mutation**			
Present	24 (6.4%)	22 (7.3%)	0.7633
Absent	351 (93.6%)	280 (92.7%)	
**APC Mutation**			
Present	291 (77.6%)	245 (81.1%)	0.304
Absent	84 (22.4%)	57 (18.9%)	
**AXIN1 Mutation**			
Present	6 (1.6%)	10 (3.3%)	0.2292
Absent	369 (98.4%)	292 (96.7%)	
**AXIN2 Mutation**			
Present	16 (4.3%)	16 (5.3%)	0.6553
Absent	359 (95.7%)	286 (94.7%)	
**CTNNB1 Mutation**			
Present	20 (5.3%)	23 (7.6%)	0.2928
Absent	355 (94.7%)	279 (92.4%)	
**GSK3B Mutation**			
Present	2 (0.5%)	5 (1.7%)	0.2516
Absent	373 (99.5%)	297 (98.3%)	
**RNF43 Mutation**			
Present	18 (4.8%)	20 (6.6%)	0.3919
Absent	357 (95.2%)	282 (93.4%)	
**TCF7L2 Mutation**			
Present	58 (15.5%)	60 (19.9%)	0.162
Absent	317 (84.5%)	242 (80.1%)	

Table S6 – Late-Onset Non-Hispanic White Patients: Treated with FOLFOX vs. Not Treated with FOLFOX

WNT Pathway			
**Gene**	**Late-Onset NHW Treated with FOLFOX n (%)**	**Late-Onset NHW Not Treated with FOLFOX n (%)**	**p-value**
**AMER1 Mutation**			
Present	71 (7.7%)	59 (9.0%)	0.4032
Absent	848 (92.3%)	594 (91.0%)	
**APC Mutation**			
Present	701 (76.3%)	478 (73.2%)	0.2007
Absent	218 (23.7%)	175 (26.8%)	
**AXIN1 Mutation**			
Present	18 (2.0%)	30 (4.6%)	**0.004453**
Absent	901 (98.0%)	623 (95.4%)	
**AXIN2 Mutation**			
Present	32 (3.5%)	64 (9.8%)	**4.44E-07**
Absent	887 (96.5%)	589 (90.2%)	
**CTNNB1 Mutation**			
Present	62 (6.7%)	38 (5.8%)	0.5239
Absent	857 (93.3%)	615 (94.2%)	
**GSK3B Mutation**			
Present	7 (0.8%)	7 (1.1%)	0.7092
Absent	912 (99.2%)	646 (98.9%)	
**RNF43 Mutation**			
Present	60 (6.5%)	96 (14.7%)	**1.48E-07**
Absent	859 (93.5%)	557 (85.3%)	
**TCF7L2 Mutation**			
Present	120 (13.1%)	118 (18.1%)	**7.79E-03**
Absent	799 (86.9%)	535 (81.9%)	

Table S7 – Early-Onset vs. Late-Onset Non-Hispanic White Patients: Treated with FOLFOX

WNT Pathway			
Gene	Early-Onset NHW Treated with FOLFOX n (%)	Late-Onset NHW Treated with FOLFOX n (%)	p-value
**AMER1 Mutation**			
Present	24 (6.4%)	71 (7.7%)	0.4764
Absent	351 (93.6%)	848 (92.3%)	
**APC Mutation**			
Present	291 (77.6%)	700 (76.2%)	0.6321
Absent	84 (22.4%)	219 (23.8%)	
**AXIN1 Mutation**			
Present	6 (1.6%)	18 (2.0%)	0.8362
Absent	369 (98.4%)	901 (98.0%)	
**AXIN2 Mutation**			
Present	16 (4.3%)	32 (3.5%)	0.6063
Absent	359 (95.7%)	887 (96.5%)	
**CTNNB1 Mutation**			
Present	20 (5.3%)	62 (6.7%)	0.4117
Absent	355 (94.7%)	857 (93.3%)	
**GSK3B Mutation**			
Present	2 (0.5%)	7 (0.8%)	1
Absent	373 (99.5%)	912 (99.2%)	
**RNF43 Mutation**			
Present	18 (4.8%)	60 (6.5%)	0.2906
Absent	357 (95.2%)	859 (93.5%)	
**TCF7L2 Mutation**			
Present	58 (15.5%)	120 (13.1%)	0.2926
Absent	317 (84.5%)	799 (86.9%)	

Table S8 – Early-Onset vs. Late-Onset Non-Hispanic White Patients: Not Treated with FOL-FOX

WNT Pathway			
**Gene**	**Early-Onset NHW Not Treated with FOLFOX n (%)**	**Late-Onset NHW Not Treated with FOLFOX n (%)**	**p-value**
**AMER1 Mutation**			
Present	22 (7.3%)	59 (9.0%)	0.4366
Absent	280 (92.7%)	594 (91.0%)	
**APC Mutation**			
Present	245 (81.1%)	478 (73.2%)	**0.01004**
Absent	57 (18.9%)	175 (26.8%)	
**AXIN1 Mutation**			
Present	10 (3.3%)	30 (4.6%)	0.4553
Absent	292 (96.7%)	623 (95.4%)	
**AXIN2 Mutation**			
Present	16 (5.3%)	64 (9.8%)	**0.0271**
Absent	286 (94.7%)	589 (90.2%)	
**CTNNB1 Mutation**			
Present	23 (7.6%)	38 (5.8%)	0.361
Absent	279 (92.4%)	615 (94.2%)	
**GSK3B Mutation**			
Present	5 (1.7%)	7 (1.1%)	0.6595
Absent	297 (98.3%)	646 (98.9%)	
**RNF43 Mutation**			
Present	20 (6.6%)	96 (14.7%)	**5.66E-04**
Absent	282 (93.4%)	557 (85.3%)	
**TCF7L2 Mutation**			
Present	60 (19.9%)	118 (18.1%)	0.5661
Absent	242 (80.1%)	535 (81.9%)	

Table S9 – Early-Onset Hispanic/Latino vs. Early-Onset Non-Hispanic White Patients: Treated with FOLFOX

WNT Pathway			
Gene	Early-Onset Hispanic/Latino Treated with FOLFOX n (%)	Early-Onset NHW Treated with FOLFOX n (%)	p-value
**AMER1 Mutation**			
Present	5 (6.8%)	24 (6.4%)	1
Absent	68 (93.2%)	351 (93.6%)	
**APC Mutation**			
Present	59 (80.8%)	291 (77.6%)	0.6495
Absent	14 (19.2%)	84 (22.4%)	
**AXIN1 Mutation**			
Present	0 (0.0%)	6 (1.6%)	0.5955
Absent	73 (100.0%)	369 (98.4%)	
**AXIN2 Mutation**			
Present	3 (4.1%)	16 (4.3%)	1
Absent	70 (95.9%)	359 (95.7%)	
**CTNNB1 Mutation**			
Present	4 (5.5%)	20 (5.3%)	1
Absent	69 (94.5%)	355 (94.7%)	
**GSK3B Mutation**			
Present	1 (1.4%)	2 (0.5%)	0.4143
Absent	72 (98.6%)	373 (99.5%)	
**RNF43 Mutation**			
Present	4 (5.5%)	18 (4.8%)	0.7687
Absent	69 (94.5%)	357 (95.2%)	
**TCF7L2 Mutation**			
Present	11 (15.1%)	58 (15.5%)	1
Absent	62 (84.9%)	317 (84.5%)	

Table S10 – Early-Onset Hispanic/Latino vs. Early-Onset Non-Hispanic White Patients: Not Treated with FOLFOX

WNT Pathway			
**Gene**	**Early-Onset Hispanic/Latino Not Treated with FOLFOX n (%)**	**Early-Onset NHW Not Treated with FOLFOX n (%)**	**p-value**
**AMER1 Mutation**			
Present	8 (15.4%)	22 (7.3%)	0.09541
Absent	44 (84.6%)	280 (92.7%)	
**APC Mutation**			
Present	44 (84.6%)	245 (81.1%)	0.6844
Absent	8 (15.4%)	57 (18.9%)	
**AXIN1 Mutation**			
Present	1 (1.9%)	10 (3.3%)	1
Absent	51 (98.1%)	292 (96.7%)	
**AXIN2 Mutation**			
Present	1 (1.9%)	16 (5.3%)	0.4853
Absent	51 (98.1%)	286 (94.7%)	
**CTNNB1 Mutation**			
Present	9 (17.3%)	23 (7.6%)	**0.04666**
Absent	43 (82.7%)	279 (92.4%)	
**GSK3B Mutation**			
Present	0 (0.0%)	5 (1.7%)	1
Absent	52 (100.0%)	297 (98.3%)	
**RNF43 Mutation**			
Present	10 (19.2%)	20 (6.6%)	**0.006037**
Absent	42 (80.8%)	282 (93.4%)	
**TCF7L2 Mutation**			
Present	11 (21.2%)	60 (19.9%)	0.9789
Absent	41 (78.8%)	242 (80.1%)	

Table S11 – Late-Onset Hispanic/Latino vs. Late-Onset Non-Hispanic White Patients: Treated with FOLFOX

WNT Pathway			
Gene	Late-Onset Hispanic/Latino Treated with FOLFOX n (%)	Late-Onset NHW Treated with FOLFOX n (%)	p-value
**AMER1 Mutation**			
Present	10 (11.0%)	71 (7.7%)	0.3729
Absent	81 (89.0%)	848 (92.3%)	
**APC Mutation**			
Present	65 (71.4%)	700 (76.2%)	0.3798
Absent	26 (28.6%)	219 (23.8%)	
**AXIN1 Mutation**			
Present	0 (0.0%)	18 (2.0%)	0.3962
Absent	91 (100.0%)	901 (98.0%)	
**AXIN2 Mutation**			
Present	3 (3.3%)	32 (3.5%)	1
Absent	88 (96.7%)	887 (96.5%)	
**CTNNB1 Mutation**			
Present	5 (5.5%)	62 (6.7%)	0.8127
Absent	86 (94.5%)	857 (93.3%)	
**GSK3B Mutation**			
Present	0 (0.0%)	7 (0.8%)	1
Absent	91 (100.0%)	912 (99.2%)	
**RNF43 Mutation**			
Present	11 (12.1%)	60 (6.5%)	0.07777
Absent	80 (87.9%)	859 (93.5%)	
**TCF7L2 Mutation**			
Present	9 (9.9%)	120 (13.1%)	0.4846
Absent	82 (90.1%)	799 (86.9%)	

Table S12 – Late-Onset Hispanic/Latino vs. Late-Onset Non-Hispanic White Patients: Not Treated with FOLFOX

WNT Pathway			
Gene	Late-Onset Hispanic/Latino Not Treated with FOLFOX n (%)	Late-Onset NHW Not Treated with FOLFOX n (%)	p-value
**AMER1 Mutation**			
Present	2 (4.0%)	59 (9.0%)	0.3011
Absent	48 (96.0%)	594 (91.0%)	
**APC Mutation**			
Present	36 (72.0%)	478 (73.2%)	0.9848
Absent	14 (28.0%)	175 (26.8%)	
**AXIN1 Mutation**			
Present	0 (0.0%)	30 (4.6%)	0.2615
Absent	50 (100.0%)	623 (95.4%)	
**AXIN2 Mutation**			
Present	1 (2.0%)	64 (9.8%)	0.07478
Absent	49 (98.0%)	589 (90.2%)	
**CTNNB1 Mutation**			
Present	2 (4.0%)	38 (5.8%)	1
Absent	48 (96.0%)	615 (94.2%)	
**GSK3B Mutation**			
Present	0 (0.0%)	7 (1.1%)	1
Absent	50 (100.0%)	646 (98.9%)	
**RNF43 Mutation**			
Present	5 (10.0%)	96 (14.7%)	0.4813
Absent	45 (90.0%)	557 (85.3%)	
**TCF7L2 Mutation**			
Present	6 (12.0%)	118 (18.1%)	0.3719
Absent	44 (88.0%)	535 (81.9%)	

Table S13 – Mutation Spectrum of WNT Pathway Genes by Ancestry, Age of Onset, and FOLFOX Treatment Status in Colorectal Cancer

	Hispanic/Latino Samples				Non-Hispanic White Samples			
	Early-Onset		Late-Onset		Early-Onset		Late-Onset	
	Treated with FOLFOX	Not Treated with FOLFOX	Treated with FOLFOX	Not Treated with FOLFOX	Treated with FOLFOX	Not Treated with FOLFOX	Treated with FOLFOX	Not Treated with FOLFOX
**AMER1**								
Frame Shift Deletion	0.0%	0.0%	30.0%	50.0%	23.1%	20.0%	22.8%	14.1%
Frame Shift Insertion	0.0%	12.5%	0.0%	0.0%	7.7%	4.0%	6.3%	8.5%
Missense Mutation	40.0%	0.0%	20.0%	50.0%	19.2%	40.0%	32.9%	40.8%
Nonsense Mutation	60.0%	87.5%	50.0%	0.0%	50.0%	36.0%	38.0%	36.6%
**APC**								
Frame Shift Deletion	33.3%	14.0%	22.8%	19.6%	30.6%	27.9%	29.9%	27.2%
Frame Shift Insertion	0.0%	10.8%	8.9%	7.1%	9.3%	8.5%	10.4%	9.8%
In Frame Deletion	0.0%	0.0%	0.0%	0.0%	0.0%	0.0%	0.1%	0.1%
In Frame Insertion	0.0%	0.0%	0.0%	0.0%	0.0%	0.0%	0.0%	0.1%
Missense Mutation	9.8%	25.8%	3.0%	1.8%	6.6%	9.0%	2.7%	6.9%
Nonsense Mutation	56.9%	46.2%	63.4%	60.7%	51.6%	52.0%	54.8%	53.7%
Silent	0.0%	0.0%	0.0%	0.0%	0.0%	0.0%	0.1%	0.0%
Splice Region	0.0%	0.0%	0.0%	1.8%	0.0%	0.0%	0.1%	0.3%
Splice Site	0.0%	3.2%	2.0%	8.9%	1.9%	2.5%	1.9%	1.9%
**AXIN1**								
Frame Shift Deletion	0.0%	0.0%	0.0%	0.0%	0.0%	8.3%	22.7%	28.6%
Frame Shift Insertion	0.0%	0.0%	0.0%	0.0%	0.0%	0.0%	4.5%	8.6%
Missense Mutation	0.0%	100.0%	0.0%	0.0%	85.7%	83.3%	63.6%	54.3%
Nonsense Mutation	0.0%	0.0%	0.0%	0.0%	14.3%	8.3%	4.5%	2.9%
Splice Site	0.0%	0.0%	0.0%	0.0%	0.0%	0.0%	4.5%	5.7%
**AXIN2**								
Frame Shift Deletion	0.0%	0.0%	33.3%	0.0%	36.8%	33.3%	25.0%	22.5%
Frame Shift Insertion	33.3%	0.0%	33.3%	0.0%	10.5%	23.8%	20.0%	22.5%
In Frame Deletion	0.0%	0.0%	0.0%	0.0%	5.3%	0.0%	5.0%	1.4%
In Frame Insertion	0.0%	0.0%	0.0%	0.0%	0.0%	4.8%	0.0%	4.2%
Missense Mutation	0.0%	100.0%	0.0%	100.0%	31.6%	33.3%	42.5%	40.8%
Nonsense Mutation	33.3%	0.0%	33.3%	0.0%	15.8%	0.0%	2.5%	2.8%
Splice Site	33.3%	0.0%	0.0%	0.0%	0.0%	4.8%	5.0%	4.2%
Translation Start Site	0.0%	0.0%	0.0%	0.0%	0.0%	0.0%	0.0%	1.4%
**CTNNB1**								
Frame Shift Deletion	0.0%	0.0%	0.0%	0.0%	0.0%	0.0%	0.0%	2.4%
In Frame Deletion	0.0%	0.0%	0.0%	0.0%	0.0%	8.3%	10.1%	2.4%
Missense Mutation	80.0%	88.9%	40.0%	50.0%	52.4%	62.5%	62.3%	78.0%
Nonsense Mutation	0.0%	0.0%	0.0%	50.0%	4.8%	4.2%	1.4%	2.4%
Nonstop Mutation	0.0%	0.0%	0.0%	0.0%	0.0%	0.0%	1.4%	0.0%
Splice Site	20.0%	11.1%	60.0%	0.0%	42.9%	25.0%	24.6%	14.6%
**GSK3B**								
Frame Shift Insertion	0.0%	0.0%	0.0%	0.0%	0.0%	4.8%	0.0%	0.0%
Missense Mutation	33.3%	0.0%	0.0%	0.0%	10.5%	14.3%	10.0%	9.9%
Nonsense Mutation	0.0%	0.0%	0.0%	0.0%	0.0%	9.5%	5.0%	1.4%
Splice Site	0.0%	0.0%	0.0%	0.0%	0.0%	0.0%	2.5%	0.0%
**RNF43**								
Frame Shift Deletion	25.0%	56.3%	37.5%	40.0%	56.5%	56.0%	50.0%	61.6%
Frame Shift Insertion	0.0%	0.0%	12.5%	20.0%	13.0%	16.0%	15.8%	8.0%
Missense Mutation	50.0%	37.5%	37.5%	20.0%	13.0%	20.0%	15.8%	17.4%
Nonsense Mutation	25.0%	6.3%	12.5%	20.0%	13.0%	4.0%	15.8%	10.9%
Splice Site	0.0%	0.0%	0.0%	0.0%	4.3%	0.0%	1.3%	2.2%
Translation Start Site	0.0%	0.0%	0.0%	0.0%	0.0%	4.0%	1.3%	0.0%
**TCF7L2**								
Frame Shift Deletion	36.4%	66.7%	22.2%	14.3%	29.5%	28.4%	25.2%	38.8%
Frame Shift Insertion	0.0%	8.3%	0.0%	28.6%	18.0%	6.0%	7.6%	7.5%
In Frame Deletion	9.1%	0.0%	11.1%	0.0%	0.0%	1.5%	1.5%	2.2%
Missense Mutation	45.5%	8.3%	55.6%	42.9%	32.8%	38.8%	37.4%	36.6%
Nonsense Mutation	0.0%	8.3%	11.1%	0.0%	13.1%	13.4%	16.0%	9.7%
Splice Site	9.1%	8.3%	0.0%	14.3%	6.6%	10.4%	11.5%	4.5%
Translation Start Site	0.0%	0.0%	0.0%	0.0%	0.0%	1.5%	0.8%	0.7%

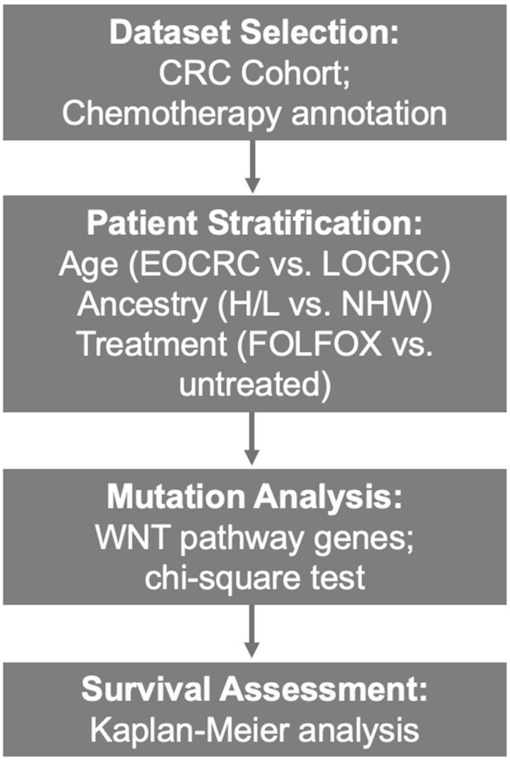

Figure S1. Study Workflow Overview. Schematic representation of the study design and analytical workflow. Clinical and genomic data were selected from CRC cohorts. Patients were stratified by age (EOCRC vs. LOCRC), ancestry (Hispanic/Latino vs. Non-Hispanic White), and treatment status (FOLFOX-treated vs. untreated). Somatic mutations in WNT pathway genes were analyzed using chi-square or Fisher's exact tests to assess differences across groups. Kaplan–Meier analyses were performed to evaluate the impact of WNT pathway alterations on overall survival, stratified by treatment exposure and ancestry.

(a)

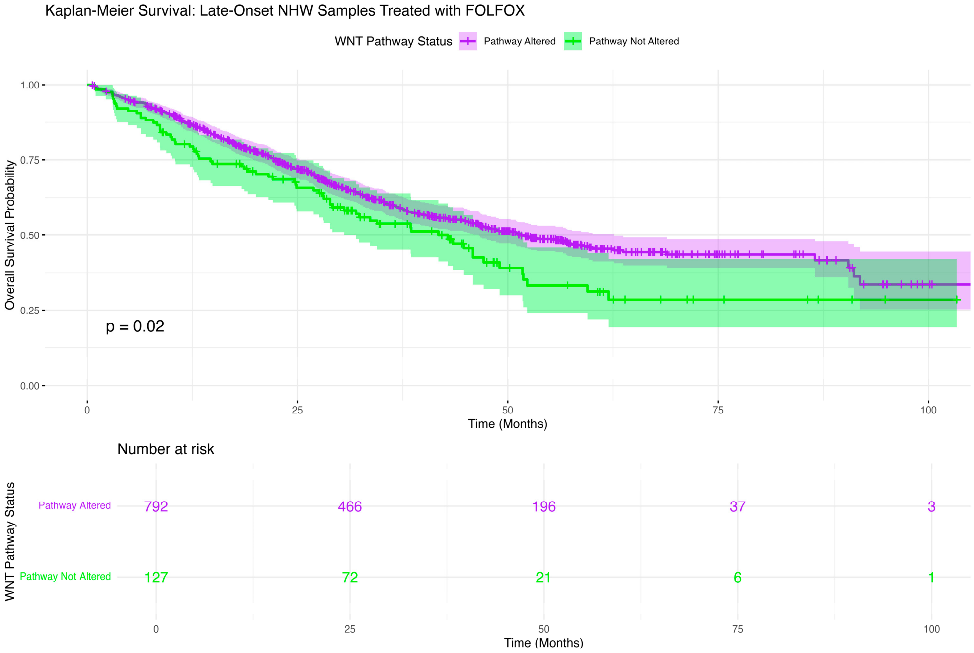

(b)

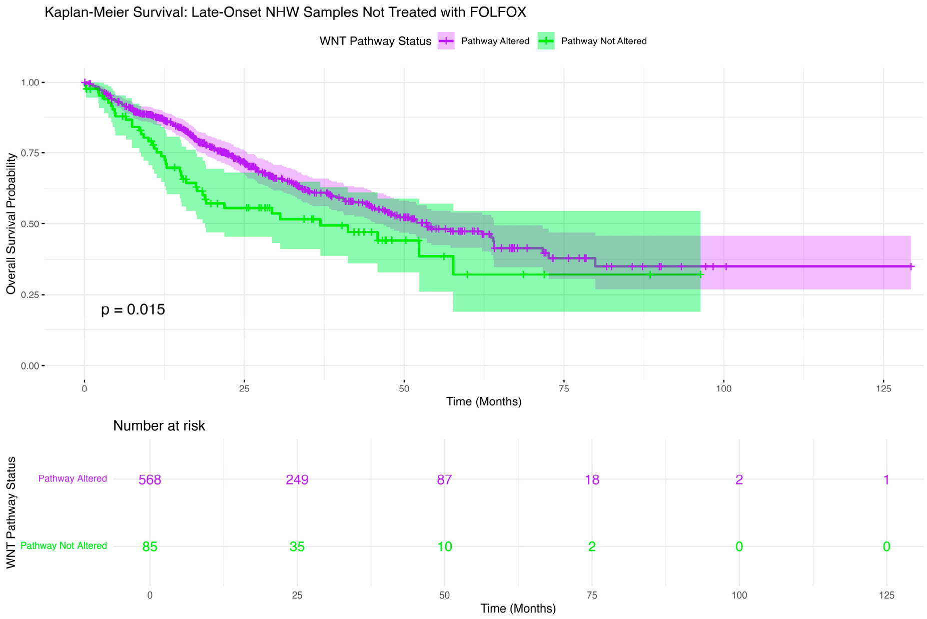

Figure S2. Comparative Somatic Mutation Landscape of WNT Pathway Genes by Age, Ethnicity, and FOLFOX Treatment Status in Colorectal Cancer. Oncoplots illustrating mutation types and frequencies across WNT pathway genes in two colorectal cancer subgroups: (a) Late-Onset NHW Treated with FOLFOX, and (b) Late-Onset NHW Not Treated with FOLFOX.
